# Living in mangroves: a syntrophic scenario unveiling a resourceful microbiome

**DOI:** 10.1186/s12866-024-03390-6

**Published:** 2024-06-28

**Authors:** Marcele Laux, Luciane Prioli Ciapina, Fabíola Marques de Carvalho, Alexandra Lehmkuhl Gerber, Ana Paula C. Guimarães, Moacir Apolinário, Jorge Eduardo Santos Paes, Célio Roberto Jonck, Ana Tereza R. de Vasconcelos

**Affiliations:** 1 Laboratório de Bioinformática, Laboratório Nacional de Computação Científica, Avenida Getúlio Vargas 333, Quitandinha Petrópolis, Rio de Janeiro, 25651-075 Brazil; 2 Petróleo Brasileiro S. A., Centro de Pesquisa Leopoldo Américo Miguez de Mello, Rio de Janeiro, RJ Brasil

**Keywords:** Metabolic reconstruction, Metagenomics, MAG, Coastal sediment, Microbial metabolism

## Abstract

**Background:**

Mangroves are complex and dynamic coastal ecosystems under frequent fluctuations in physicochemical conditions related to the tidal regime. The frequent variation in organic matter concentration, nutrients, and oxygen availability, among other factors, drives the microbial community composition, favoring syntrophic populations harboring a rich and diverse, stress-driven metabolism. Mangroves are known for their carbon sequestration capability, and their complex and integrated metabolic activity is essential to global biogeochemical cycling. Here, we present a metabolic reconstruction based on the genomic functional capability and flux profile between sympatric MAGs co-assembled from a tropical restored mangrove.

**Results:**

Eleven MAGs were assigned to six Bacteria phyla, all distantly related to the available reference genomes. The metabolic reconstruction showed several potential coupling points and shortcuts between complementary routes and predicted syntrophic interactions. Two metabolic scenarios were drawn: a heterotrophic scenario with plenty of carbon sources and an autotrophic scenario with limited carbon sources or under inhibitory conditions. The sulfur cycle was dominant over methane and the major pathways identified were acetate oxidation coupled to sulfate reduction, heterotrophic acetogenesis coupled to carbohydrate catabolism, ethanol production and carbon fixation. Interestingly, several gene sets and metabolic routes similar to those described for wastewater and organic effluent treatment processes were identified.

**Conclusion:**

The mangrove microbial community metabolic reconstruction reflected the flexibility required to survive in fluctuating environments as the microhabitats created by the tidal regime in mangrove sediments. The metabolic components related to wastewater and organic effluent treatment processes identified strongly suggest that mangrove microbial communities could represent a resourceful microbial model for biotechnological applications that occur naturally in the environment.

**Supplementary Information:**

The online version contains supplementary material available at 10.1186/s12866-024-03390-6.

## Background

Mangroves are complex and dynamic coastal and estuarine ecosystems composed of mangrove trees and associated communities of populations specialized to thrive in the transition between land and sea under frequent fluctuations in physicochemical conditions promoted by the tidal regime (the rise and fall of the sea level) [1–7]. The tidal influence may be seasonal or daily [8, 9], and the sediment composition and properties greatly vary even at very short distances [10–12]. Under such unstable conditions, microbial communities with populations harboring different and complementary metabolic capabilities engaged in tightly balanced syntrophic relationships are advantageous [3, 11, 13, 14].

The periodic tidal flooding and drainage results in variable organic matter and nutrients concentration, alkaline conditions with high salinity, high sulphate concentration, unstable redox conditions, and prevalence of anoxic conditions, directly driving the microbial community composition [1, 2, 4, 15–18]. In high tide, the seawater brings sulphate ions, and infiltrate in the interstitial sediment. When the water level starts to fall, in the transition between the high and low tides, it flushes out nutrients and organic matter from the carbon-rich deep porewater, favoring microbial populations able to grow autotrophically. During the transition to low tide, the porewater is less mobile, and the dissolved oxygen progressively decreases, and the sulphate deposition favor the increase in abundance of sulfate-reducing bacteria (SRB) populations. During low tide, the static old porewater becomes anoxic, and nutrients and carbon are retained, increasing the organic matter and carbon sources availability, creating a carbon-rich microhabitat favorable to microbial populations capable of oxidate a variety of substrates through anaerobic processes [4, 19].

Mangrove ecosystems are considered ‘Blue Carbon Ecosystems’ due to their carbon storage capability [20, 21], and the diverse and integrated metabolic activity is essential to global biogeochemical cycling [4, 6, 11, 18]. Among the mangrove ecosystem services are the ability to break down vegetal biomass [18, 22, 23], recycling of a variety of nutrients, carbon sources and xenobiotics [4, 24–26], adaptations to osmotic stress and redox gradients [2, 17, 27, 28], and resistance to metals and chemicals [27, 29–31]. Such a rich metabolic context is valuable for discovering unculturable and rare populations harboring key metabolic pathways and enzymes involved in stress response and transformation processes.

The advent of environmental DNA (eDNA) methods such as metagenomics has been a driving force of biodiversity discovery, allowing the investigation of unknown uncultured organisms and providing access to the genetic content of whole communities from any ecosystem [7, 32]. Most mangrove studies using eDNA have explored, for example, the impacts of anthropogenic contamination [27, 29–31, 33], detailed investigations on methane, nitrogen and sulfur cycling genes/pathways [4, 11, 34], enzymes with potential use in biotechnological processes [15, 16, 22, 23, 25, 26, 30, 35], community responses to global warming and sea level rising [5, 12, 21, 36], and microbial diversity of mangroves from distinct biomes to micro-habitats [12, 37].

The development of assembly and binning tools has allowed eDNA studies to evolve from gene-centric methods to genome-resolved metagenomics [38]. The large volume of data generated by next-generation sequencing technologies has enabled the pool of short sequence reads to be assembled into contigs and binned into metagenome-assembled genomes (MAGs) [39], leading to the genome reconstruction of well-established species in parallel to uncultured taxa [38, 40]. Reconstruction of MAGs from mangrove ecosystems has led to the discovery of novel microbial community members and key functional pathways [14, 26], as well as investigations about microbial synergism, syntrophic relationships, and coupling biogeochemical processes [11, 13, 38]. The syntrophic interactions between community members sharing metabolite exchange are essential for the development and maintenance of microbial ecosystems [41–43]. In mangroves, changes in the microbial community structure and syntrophic functional dynamics have been better understood through genome-scale metabolic models (GEM), flux balance analysis (FBA) and network analysis, as demonstrated in recent studies [11, 13].

This study describes the metabolic reconstruction of sympatric MAGs assembled from a tropical restored mangrove adjacent to an oil refinery, providing insights into community-level syntrophic scenarios through complementary metabolic routes. To search for the possible connections and potential coupling mechanisms, we mapped the genes and reactions of the most representative carbon and energy metabolism pathways. The genome-scale metabolic models and flux balance analysis were applied to verify metabolic activity and exchange within the ETDI mangrove microbial community.

## Methods

### Sample collection and sequencing

Nine sediment samples from a restored mangrove adjacent to an oil refinery in a region named ETDI, which is an effluent treatment station, located in the “Baía de Todos os Santos”, state of Bahia, Brazil (-12.7105 –38.5650 W), were collected from 4 to 10 October 2021, in the transition between high and low tide at 0 to 2 cm depth. The predominant vegetation was *Rhizophora mangle* with the presence of *Avicennia schaueriana* and *Laguncularia racemosa*. Soil samples were collected with a sterilized stainless steel spatula, with the collector wearing a face mask and nitrile gloves. Soils were placed in RNase-free Falcon tubes and kept on ice (4 °C) until arrival at the field base. There, they were frozen at − 20 °C and later transported on dry ice to the laboratory, where they were stored in an ultrafreezer (− 80 °C) until processing. Total DNA was extracted using the PowerSoil^®^ DNA Isolation Kit (Mobio Labs, Inc., Solana Beach, CA, USA) at SENAI Institute of Innovation in Biosynthetic and Fibers (SENAI CETIQT, Rio de Janeiro, RJ, Brazil). Metagenomics libraries were constructed using the Nextera DNA Flex Library Prep Kit (Illumina) according to the manufacturer’s protocol. Sequencing was performed on an Illumina NextSeq 500 platform (2 × 150 bp) (Illumina, San Diego, CA) at Computational Genomics Unity Darcy Fontoura de Almeida (UGCDFA) of the National Laboratory of Scientific Computation (LNCC) (Petrópolis, RJ, Brazil). Based on the total length of the high and medium-high quality MAGs assembled, the ETDI samples sequencing depth was 41x on average. Details about geographic localization, characteristics of sampling sites and metrics can be found in the related paper of our team Carvalho and collaborators (2024) [44].

### MAGs workflow

The metagenome assembly, binning, and genome quality control were carried out by the *System for Automated Bacterial Integrated Annotation – Sabiá* [45]. For the reconstruction of individual genomes from metagenomic data (MAGs), the “co-assembly” approach was used to maximize the number of recovered MAGs. The coverage values of each contig were calculated from the individual samples, according to the approach described by [46]. Metagenome assembly was performed using Megahit software [47], with a minimal contig length of 200, meta-large presets, and kmer values between 27 and 127. Contigs greater than 2,500 bp were submitted to the binning step by Metabat2 software [46] (default parameters). Potential MAGs underwent quality control using the CheckM software [48]. Quality control was based on completeness and contamination estimates, according to the minimum information about a metagenome-assembled genome (MIMAG) criteria [49]. ETDI MAGs with completeness ≥ 50.0 and contamination ≤ 10.0% were selected for the subsequent taxonomic assignment and functional annotation steps. The taxonomic identification of the MAGs was performed using the GTDB-Tk software [50] (default parameters). The open reading frames (ORFs) were predicted using Prodigal software [51] and the functional assignment was based on the alignment of the identified ORFs against the NCBI NR database [52], and EggNOG database [53], which compiles information from COG [54], KEGG Orthology (KO) [55], Gene Ontology (GO) [56], CaZY [57], and PFAM database [58].

### Metabolic reconstruction

ETDI MAGs with high- and medium-high quality, that is, with completeness ≥ 90.0 and contamination ≤ 10.0% [49], were selected for the subsequent analysis. The metabolic reconstruction was based on the presence/absence of ORFs assigned to KEGG KO in each MAG. To describe the individual and shared metabolic features, we considered those with the highest completeness of KO’s assigned as the most representative pathways. We prioritized the complete pathways/metabolic routes (presenting all required genes and reactions) and selected only those whose sequence of reactions and MAGs involved could be potentially functional according to the environmental context and scientific literature, which will be detailed below. To describe a possible syntrophic scenario, we search for the potential coupling mechanisms and complementary pathways based on a careful manual curation according to the scientific literature.

The genome-scale models (GEMs) are a network-based strategy that uses all available information about gene-protein-reaction associations to reconstruct an organism’s metabolism [59]. The eleven GEMs were built using the MS2 - Build Prokaryotic Metabolic Models app in the Kbase platform [60], which is based on the ModelSEED Pipeline for genomes annotated using the RAST functional ontology and biochemistry database [61, 62]. The Flux Balance Analysis (FBA) was performed by the Run Flux Balance Analysis app [63]. This app analyzes the organism’s growth on different substrates and enables it to evaluate the reactions and metabolites that carry fluxes in each growth condition [63]. The Edit Media App was used to create different media formulations by adding or removing compounds and modifying compound concentrations. The media that produced the higher objective value (model growth) were selected for analyzing the flux profile and exchange fluxes. According to the Kbase pipeline, the ‘complete’ media consists of all metabolites for which a transporter is available in the KBase biochemistry database [60]. Consequently, the complete media does not present an exact list of compounds [60]. The C-D-Glucose minimal media (refglumin) was selected to represent the reactions and compound exchanges occurring when little substrate is available. The C-D-Lactose media was modified by removing the lactose and adding a high concentration of CO2 to simulate the autotrophic conditions. All media were tested both with and without oxygen. The KBase FBA pipeline can be accessed through a static narrative in the following link: 10.25982/157568.747/2335480.

### Phylogenomic analyses

The phylogenomic analyses were conducted considering the high and medium-high quality MAGS. The evolutionary inference of each MAG was evaluated separately, through comparison with taxa belonging to the taxonomic hierarchical level above to which the respective MAG was classified and whose genomes were publicly available in the NCBI database. The steps after obtaining the genomes were carried out according to the phylogenomics workflow described by Graham et al. (2018) [64]. The coding genes prediction was initially performed using the Prodigal program, using default parameters. Then, the HMMSEARCH of the HMMER program (http://hmmer.org/) was applied to obtain a central panel of marker ribosomal proteins, useful for attributing MAGs. The alignment, cutting and concatenation of proteins were performed using, respectively, the software MUSCLE [65], TrimAL [66], and CONCAT (script on BinSanity package) [67], all with default parameters. The phylogenetic tree was then generated using FASTTREE [68], and -gamma and -lg parameters as models for calculating branch length and amino acid evolution, respectively). The LG model [68] was applied to verify the rate of evolution of amino acids. Rates of evolution between sites were estimated by CAT approximation with 20 rate categories. Bootstrap values, ranging from 0 to 1, are presented in each branch. Species of the genus *Bacillus* (phylum Firmicutes) were used as an outgroup for rooting the tree. The phylogenomic reconstruction was then annotated using the iTOL tool [69]. Additional workflow details are available at https://github.com/edgraham/PhylogenomicsWorkflow.

## Results

### Phylogenomics

From a total of 27 metagenome-assembled genomes (MAGs) recovered from ETDI metagenome datasets, 16 presented medium-quality ( > = 50% completion and < 10% contamination), two presented medium-high quality (> 90% completion, < 7% contamination), and nine presented high-quality (> 90% completion, < 5% contamination) [49] (Table S1). According to the taxonomic inference generated by the GTDB database, the 11 MAGs of medium-high and high quality were assigned to six phyla, four classes, four orders, two families, and one known genus (Table 1). The Phycisphaerae (PY) MAG presented the largest genome (5.2Mbp), followed by Desulfobacterales (DE) (4.7Mbp) and Acidobacteria (AC) (4.5Mbp). The Porticoccaceae (PO), and the Alcanivoracaceae (AL) MAGs presented the smallest genomes (2.66Mbp and 2.6Mbp, respectively). The highest coding density was observed in AC, which also showed the highest GC content (0.71). Detailed assembly metrics of each MAG are presented in Table S1.

**Table 1:** Taxonomic assignment, completeness and contamination and genome coverage of the eleven high quality MAGs

	CC	Cov	Phylum	Class	Order	Family	Genus
**PS11**	90.2/3.5	9x	Proteobacteria	Gammaproteobacteria	Pseudomonadales	-	-
**PS82**	98/0.7	31x	Proteobacteria	Gammaproteobacteria	Pseudomonadales	UBA3067	-
**AL**	96.6/0.7	15x	Proteobacteria	Gammaproteobacteria	Pseudomonadales	Alcanivoracaceae	PGZG01
**PO**	95.7/3.6	61x	Proteobacteria	Gammaproteobacteria	Pseudomonadales	Porticoccaceae	HB23221
**TH88**	97/3.7	8x	Proteobacteria	Gammaproteobacteria	Thiohalomonadales	UBA6429	UBA6492
**TH94**	94/2.6	23x	Proteobacteria	Gammaproteobacteria	Thiohalomonadales	UBA6429	-
**DE**	96.8/2.4	11x	Desulfobacterota	Desulfobacteria	Desulfobacterales	-	-
**SU**	95.4/5.4	7x	Nitrospirota	Thermodesulfovibrionia	Thermodesulfovibrionales	UBA6898	Sulfobium
**PY**	92/4.5	13x	Planctomycetota	Phycisphaerae	UBA1845	UBA1845	-
**ZI**	100/2.2	9x	Zixibacteria	MSB-5A5	CAIYYT01	CAIYYT01	-
**AC**	91.7/6.9	15x	Acidobacteriota	Mor1	Mor1	-	-

PS11: Pseudomonadales ETDI_bin11, PS82: Pseudomonadales ETDI_bin82, AL: Alcanivoracaceae ETDI_bin14, PO: Porticoccaceae ETDI_bin28, TH88: Thiohalomonadales ETDI_bin88, TH94: Thiohalomonadales ETDI_bin94, DE: Desulfobacterales ETDI_bin60, SU: Sulfobium ETDI_bin26, PY: Phycisphaerae ETDI_bin83, ZI: Zixibacteria ETDI_bin71, AC: Acidobacteria ETDI_bin10. CC: completeness/contamination, Cov: genome coverage

**Fig. 1:**
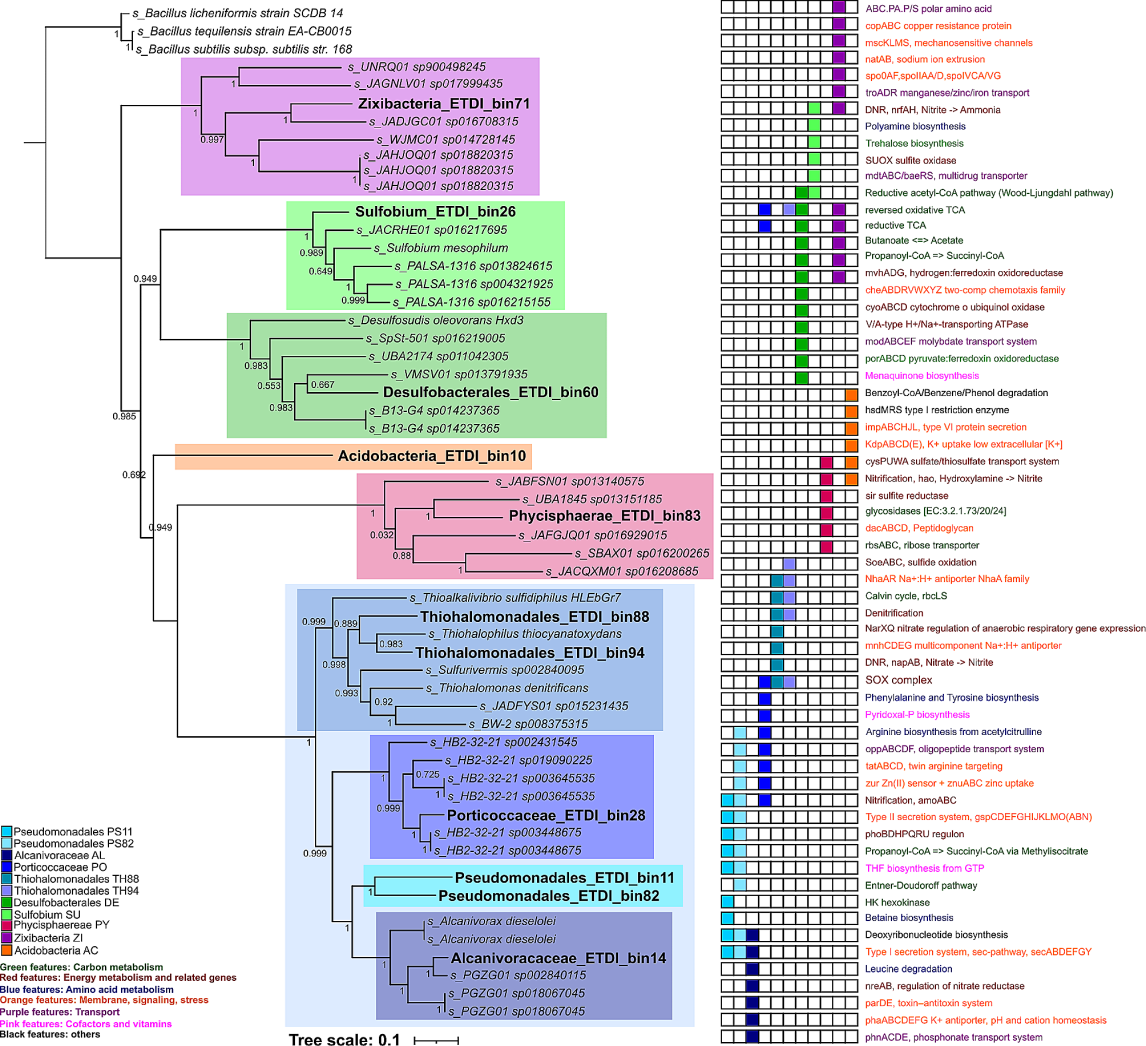
Phylogenomic reconstruction of the 11 ETDI MAGs and their evolutionary relationships with public reference genomes. Colored boxes represent the occurrence of that pathway/route/protein in the respective MAG

Most MAGs belonged to the phylum Proteobacteria, Gammaproteobacteria class, known as abundant members of tropical mangroves [1, 3–5, 7, 37, 70, 71], and showed phylogenomic proximity to reference genomes from taxa with little information in the literature. Members of the class Gammaproteobacteria were placed together within the same clade, where the two members of the order Thiohalomonadales (TH88, TH94) and the four members of the order Pseudomonadales (PS11, PS82, PO and AL) were positioned in separated sub-clades (Fig. 1). The MAG assigned to the Phycisphaerae class (PY) was positioned in a distinct clade, closer to Gammaproteobacteria. The MAGs assigned to the order Desulfobacterales (DE) (c_Desulfobacteria) and to the genus *Sulfobium* (SU) (c_Thermodesulfovibrionia; o_hermodesulfovibrionales) were positioned within the same clade, more distant from Gammaproteobacteria, as also observed for the MAG affiliated with the phylum Zixibacteria (ZI) (Fig. 1). Monophyletic clades were observed for the Acidobacteria (AC) MAG, and the two Pseudomonadales (PS11, PS82) MAGs (Fig. 1), showing that the genomes possess a considerable genomic heterogeneity compared to the public genomes, and are potential new taxonomic members.

Previous studies in mangrove systems from Brazilian cost described Proteobacteria (AL, TH, PO), and Planctomycetes (PY) among the most abundant Phyla [1, 4, 5, 7, 8, 22, 70, 72, 73], while Desulfobacteria (DE) was associated to sulfate reduction, low oxygen, and oil impacted mangrove area [7, 15, 30, 31]. Nitrospirota phylum (SU) and Acidobacteria (AC) were detected among the less abundant taxa [1, 5, 7, 31, 33, 72]. Zixibacteria (ZI) was not previously reported in Brazilian mangrove systems and has been described as highly versatile, with high co-occurrence rates and connectivity in mangrove systems [12, 13, 74].

### Living in mangroves

**Fig. 2:**
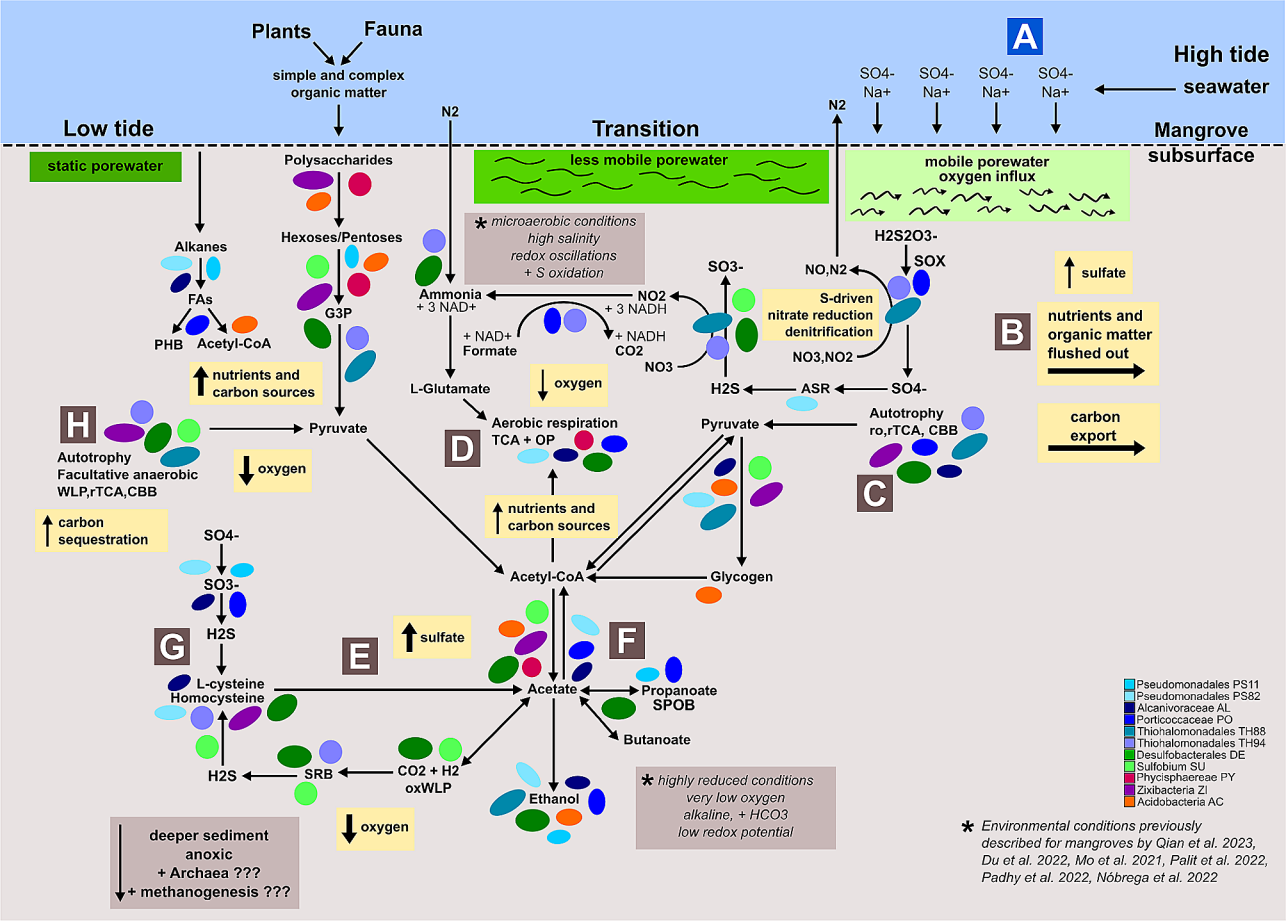
General overview of mangroves major pathways identified among the eleven high and medium-high ETDI MAGs. Circles and ellipses represent occurrences of each MAG in each route or step. This figure represents the metabolism as a whole, not schematized under the determination of space and time factors. **A**-**H** letters represent guides for a better interpretation of the interconnection between the steps and processes of metabolic dynamics occurring in the community according to the references as indicated in the bottom right

In this section is presented the overall mangrove metabolism reconstruction based on previous studies, which is illustrated in Fig. 2 (A-H letters represent guides for a better interpretation of the metabolic flow described in the text below). A total of 3644 KOs were annotated for the 11 ETDI MAGs, which together formed 122 complete KEGG modules, mostly related to amino acid metabolism (29), carbohydrate (23), metabolism of cofactors and vitamins (20), and energy (19). The KEGG pathways and modules distribution among the MAGs are presented in Table S2 and Table S3, respectively. Considering the 11 high and medium-high quality assembled MAGs as representatives of the most abundant populations at the time of sampling, metabolic components of aerobic and anaerobic metabolism were observed, the pathways related to facultative anaerobic metabolism were predominant and the sulfur cycle was dominant over methane. The most representative pathways previously described for mangrove ecosystems were identified among the MAGs, including the degradation of complex organic matter, carbon sequestration, nutrient cycling, sulfur transformation, and adaptations to osmotic and oxidative stress, essential for the electrochemical stability [3, 4, 11, 13, 40]. As will be further described in the following sections, two simultaneous carbon metabolism scenarios driven by the tidal dynamics were drawn: heterotrophic scenario, with plenty of carbon sources similar to environmental conditions described for low tide, and autotrophic scenario, with carbon sources limited or under inhibitory conditions, similar to high tide and transition from high tide to low tide conditions. As illustrated in Fig. 2, during high tide, the seawater influx brings sodium, chloride and sulfate ions, which infiltrate in the interstitial sediment water (porewater) [4, 9, 18] (Fig. 2a). The superficial porewater is mobile and constantly flowing, and drastically changes its physical and chemical conditions as the depth increases [4]. When the water level starts to fall, in the transition between the high and low tides, it flushes out nutrients and organic matter from the carbon-rich deep porewater, potentially creating carbon and nutrient limiting conditions resulting from the carbon export [4, 18] (Fig. 2b). During this carbon and nutrient limiting period, microbial populations able to grow autotrophically would increase in abundance, reestablishing the microhabitat conditions required by heterotrophic populations [40] (Fig. 2c). During the transition to low tide, the porewater is less mobile, the dissolved oxygen progressively decreases, and nutrients and organic carbon progressively increase, resulting from carbohydrates breakdown and oxidation and heterotrophic microbial respiration [4, 13, 18] (Fig. 2d). In such tidal microhabitats, the sulfate deposition would favor the increase in abundance of SRB, which grow by sulfate reduction to sulfide, coupled with oxidation of hydrogen or organic compounds [34, 70] (Fig. 2e). The electrochemical conditions may be balanced by volatile fatty acids (VFA) production and oxidation (Fig. 2f) and sulfide biotransformation strategies [34] (Fig. 2g). During low tide, the static old porewater may retain nutrients and carbon. In this condition, the intense microbial activity may cause a decrement in oxygen and an increase in the CO2 and H2 concentration, creating a carbon-rich microhabitat that may inhibit the heterotroph’s glycolytic activity [3, 4, 9]. Under this inhibitory condition, the facultative anaerobic autotrophs would be favored and could be involved in carbon sequestration [3, 18, 75] (Fig. 2h). In the scenarios described here, the flux predictions and exchange of compounds occurring among the pathways were validated by the FBA analysis, with a nonzero mass-balanced flux, corroborating the KEGG reconstruction.

### Sulfur metabolism

**Fig. 3:**
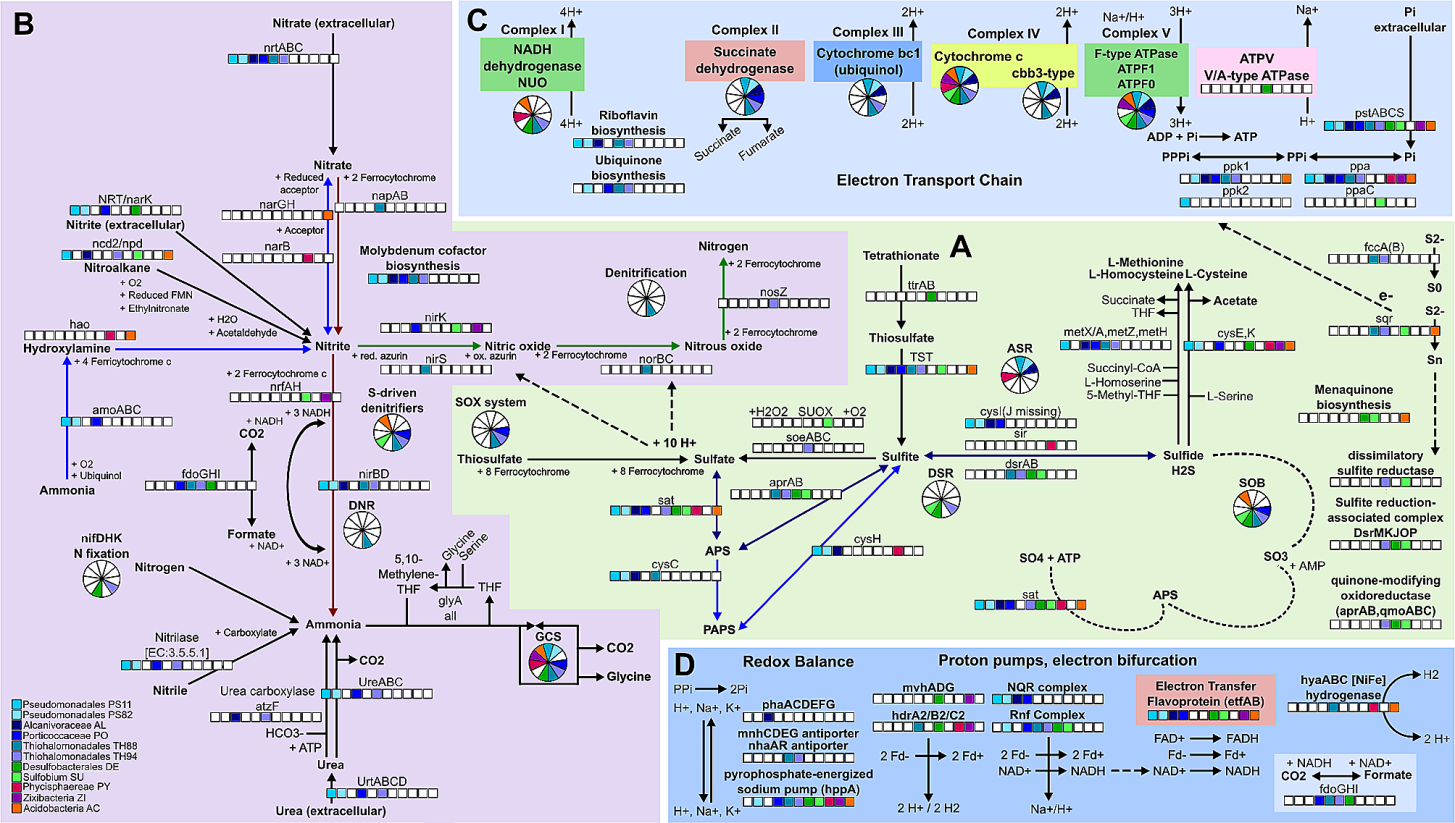
Energy metabolism. **A**: sulfur metabolism, **B**: nitrogen metabolism, **C**: ATP synthesis, **D**: proton pumps

Among the mangrove common bio-geochemical cycles, the sulfur cycle is one of the major contributors to organic matter mineralization [70, 76, 77]. Interestingly, the sulfur cycle was dominant over methane at the time of sampling. We assumed this statement since we could not find enough genes required for methanogenesis and methanotrophy to consider the pathway/route present in the eleven MAGs, as well as in the low quality MAGs, in the metagenome dataset, and in other samples from this project. We suppose that this finding is probably related to the tidal regime, responsible for high salinity and sulfate concentration, among other factors that favor SRB and sulfur-oxidizing bacteria (SOB) populations [78, 79]. The sulfate respiration can occur via the dissimilatory sulfate reduction pathway (DSR), characteristic of SRB, and the Apr-QmoABC complex, linked to the menaquinone pool [80]. The gene set of sulfate respiration by DSR (M00596) and by the Apr-QmoABC complex was identified in TH88, TH94, DE and SU MAGs (Fig. 3a; Table 2). The presence of DSR genes in these MAGs is expected since members of Desulfobacterales [27, 34, 72, 81], and Thermodesulfovibrionales [82–84] orders are known as SRB. The enrichment of genes related to sulfate reduction were previously described in Brazilian mangroves [4, 7, 8, 30, 31, 72]. SRB activity is strongly dependent on several environmental factors, with genes working in both reductive and oxidative directions [34, 78]. Although the presence of DSR genes per se does not confirm SRB activity, flux was predicted in DE and TH88 (Table 3). When active, sulfate respiration produces massive amounts of the highly toxic reduced sulfide (hydrogen sulfide), the end-product of DSR, which impacts may be balanced by the co-occurrence of SRB and populations harboring sulfide biotransformation genes and complexes [14, 34, 76, 77]. Table 3 presents the FBA profile of the main energy and glycolytic pathways described for the complete (CO), customized aerobic (CA), customized anaerobic (CN), autotrophic aerobic (AA) and autotrophic anaerobic (AN) media.

**Table 2:** Main genes, operons and complexes identified according to the KEGG reconstruction for each MAG

PS11	PS82	AL14	PO28	TH88	TH94	DE60	SU26	PY83	ZI71	AC10	Module/Gene set/Operon/Complex	Pathway/Roles
				X	X	X	X				Dissimilatory sulfate reduction DSR, sulfate => H2S, M00596, AprAB/QmoABC	Sulfur/SRB
				X	X						fccA cytochrome subunit of sulfide dehydrogenase	Sulfur metabolism/SOB
				X	X		X			X	sqr sulfide: quinone oxidoreductase	Sulfur metabolism/SOB
X	X	X	X	X		X	X	X		X	sat met3 sulfate adenylyltransferase [EC:2.7.7.4]	Sulfur metabolism/SRB/SOB
X	X		X			X		X	X	X	cysEK, L-Cysteine biosynthesis	Sulfur metabolism/AA
X		X	X	X	X						metAX, metBZ, L-Homocysteine biosynthesis	Sulfur metabolism/AA
						X					dmsBC dimethyl sulfoxide reductase (alpha missing)	Sulfur/DMSO respiration
			X	X	X			X			tsdA thiosulfate dehydrogenase [EC:1.8.2.2]	Sulfur metabolism/SOB
						X				X	psrA thiosulfate reductase/polysulfide reductase chain A [EC:1.8.5.5]	Sulfur metabolism/SOB
X	X	X	X	X	X						TC.SULP sulfate permease SulP family	Sulfate uptake/inorganic anions
			X				X		X		nirK nitrite reductase (NO-forming) [EC:1.7.2.1]	Nitrogen metabolism
						X					cyoABCD, cytochrome o ubiquinol oxidase, M00417	ATP synthesis
	X			X	X	X	X	X	X	X	PYG glgP glycogen phosphorylase [EC:2.4.1.1]	Glycogen degradation
	X			X	X					X	malQ 4-alpha-glucanotransferase [EC:2.4.1.25]	Glycogen degradation
										X	glgX glycogen debranching enzyme [EC:3.2.1.196]	Glycogen degradation
							X	X		X	ISA iamylase [EC:3.2.1.68], AMY [EC:3.2.1.1] glycogen => maltose + dextrin	Glycogen degradation
				X			X				treS alpha-amylase [EC:5.4.99.16 3.2.1.1] glycogen => maltose + dextrin	Glycogen degradation
	X			X	X	X	X			X	glgABC glucose-1P => amylose => glycogen	Glycogen biosynthesis
						X				X	PFP Pii-dependent phosphofructokinase, D-Fructose-6P <=> D-Fructose-1,6Pii	Glycolysis alternative
						X	X		X	X	ppdK pyruvate orthophosphate dikinase [EC:2.7.9.1]	Glycolysis alternative
				X	X						PRK+rbcLS Calvin cycle incomplete	CBB/Carbon fixation
		X			X	X	X		X		PGAM gpmA 2,3-bisphosphoglycerate-dependent phosphoglycerate mutase [EC:5.4.2.11]	CBB=> glycolysis via glycerate-3P
X	X		X	X	X	X				X	gpmI 2,3-bisphosphoglycerate-independent phosphoglycerate mutase [EC:5.4.2.12]	CBB=> glycolysis via glycerate-3P
							X	X			apgM 2,3-bisphosphoglycerate-independent phosphoglycerate mutase [EC:5.4.2.12]	CBB=> glycolysis via glycerate-3P
X			X				X	X	X		rbsK+rpiA/B+rpe: D-Ribose => D-Ribulose-5P => D-Xylulose-5P	Pentoses oxidation
						X		X			xylAB: D-Xylose => D-Xylulose => D-Xylulose-5P	Pentoses oxidation
				X	X			X		X	xfp_xpk+ackA: D-Xylulose-5P => Acetate	Pentoses oxidation
X	X	X	X	X	X	X	X	X	X		TPI+gpsA: G3P <=> glycerone-P <=> glycerol-3P	Glycolysis to Glycerophospholipid metabolism
X	X	X		X	X	X					glpK+gpsA/glpA: Glycerol <=> glycerol-3P <=> glycerone-P	Glycerol utilization
	X			X	X				X	X	ppc phosphoenolpyruvate carboxylase [EC:4.1.1.31]	rTCA key enzimes
X			X		X						pycAB pyruvate carboxylase subunit A [EC:6.4.1.1]	rTCA key enzimes
						X					PC pyc pyruvate carboxylase [EC:6.4.1.1]	rTCA key enzimes
X						X				X	E4.1.1.32 pckA PCK phosphoenolpyruvate carboxykinase(GTP)[EC:4.1.1.32]	rTCA key enzimes
X	X	X	X					X	X		E4.1.1.49 pckA phosphoenolpyruvate carboxykinase(ATP)[EC:4.1.1.49]	rTCA key enzimes
				X							PEPCK phosphoenolpyruvate carboxykinase(diphosphate)[EC:4.1.1.38]	rTCA key enzimes
										X	citEG citrate lyase, Citrate <=> Acetate + Oxaloacetate (gamma missing)	rTCA key enzimes
				X	X						IclR family transcriptional regulator acetate operon repressor	VFAs/Acetate metabolism
			X				X	X		X	actP cation/acetate symporter	VFAs/Acetate metabolism
						X	X		X	X	Acetate---CoA ligase acdAB	VFAs/Acetate metabolism
						X					ACH1 acetyl-CoA hydrolase	VFAs/Acetate metabolism
X	X	X	X		X	X	X	X	X	X	bifunctional Acetyl-CoA synthetase ACSS1_2/acs	VFAs/Acetate metabolism
X	X	X	X	X	X	X	X	X	X	X	ACAT atoB acetyl-CoA C-acetyltransferase [EC:2.3.1.9]	VFAs/Acetate metabolism
						X					atoAD acetate CoA/acetoacetate CoA-transferase	VFAs/Acetate metabolism
		X		X	X	X			X		wrbA NAD(P)H dehydrogenase(quinone) [EC:1.6.5.2]	Propanoate metabolism
						X					E2.1.3.1–5 S,1.3 S,12 S (S)-Methylmalonyl-CoA+Pyruvate<=> Propanoyl-CoA+Oxaloacetate	Propanoate metabolism
						X					pccAB Propanoyl-CoA+HCO3-<=> (S)-Methylmalonyl-CoA	Propanoate metabolism
									X	X	mcmA1/A2 (R)-Methylmalonyl-CoA <=> Succinyl-CoA	Propanoate metabolism
X	X	X									prpCFB Propanoyl-CoA<=> Pyruvate + Succinate via 2-Methylcitrate	Propanoate metabolism
X	X		X								acnDB Propanoyl-CoA<=> Pyruvate + Succinate via 2-Methylcitrate	Propanoate metabolism

**Table 3:** FBA profile showing the predicted flux through the main energy and glycolytic pathways

	PS11	PS82	AL	PO	TH88	TH94	DE	SU	PY	ZI	AC
SRB					AAN, CAN, CP		AAN, CP				
SO4 reduction to SO3 (cytochrome)		AN, CN	AN	AN				AN	AN, CN	AAN	AN, CN
SO4 reduction to APS	AAN	CN	AA		AAN, CAN	AN, CAN	AAN	AAN, CAN	AA		AA
DSRox					CO	CAN, CP, CO	CAN, CO	CN, CO			
H2S => SO3					AAN, CAN, CO	CA, CO	AAN, CAN, CO	CA, CO			
SOX				CAN, CO	CAN	CA					
H2S => L-Cysteine/L-Homocysteine	AAN, CAN, CO	AAN, CAN, CO	AAN, CAN, CO	AAN, CAN, CO	AAN, CAN, CO	AAN, CAN	AAN, CAN		AAN, CAN	CAN, CO	AAN, CAN
DMSO		CO		CO							
Sulfate uptake/ATP hydrolysis	AN						AN	CN	AN, CN		AN, CN
Nitrification1	AN					AN	AA, AN		AN		AN
Nitrification2							AA				
DNR1 (NO4 => NO3)		CAN, CO	CN	CAN	CN	CA, CO					CN
DNR2 (NO3 => NH3)	CA	CAN, CO	CAN	CAN	CAN	CAN, CO	AN, CAN		CN		CAN
Denitrification2		AA		AA, AN, CA, CN		AA, CA, CN	AA	AA		AA, AN	
Denitrification3						CA	CO	CO	CO	CN, CO	CO
Denitrification4					CA, CN, CO						
formate => CO2 (NADH)	CAN			AAN, CAN		AAN, CAN	CAN	AA, CA	CAN		AA
N fixation					CAN, CO		CO	CO			
Urea hydrolysis	AA	CAN		AAN, CAN, CO	AAN, CAN						
NH3 => Nitrite	AA	AAN	AAN	AAN	AAN	AA	AN	AA	AA	AAN	AA
NH3 => L-Glutamate		AA, CN	CN	CAN	AAN				AAN, CA, CO	AAN, CAN, CO	AAN, CN
S-driven DNR/denitrification				CAN	CAN	CA, CO		CO			
OP complex II	CO	AAN, CA	CAN, CO	AAN, CAN, CO				AAN, CAN, CO	CAN, CO		CAN, CO
OP complex III			AAN, CA, CO				CA, CO	AAN, CAN	AA, CA, CO	AAN, CAN, CO	AAN, CAN, CO
OP complex IV	AAN, CAN, CO	AAN, CAN	AAN, CAN	AAN, CAN, CO	CN	AAN, CAN, CO		AAN, CAN, CO	AAN, CAN, CO	AAN, CAN	AN, CN
RNF complex	AN				AN, CA	AN					
Glycogen => Glucose-1P		CN									
Maltose => D-Glucose					CAN	CAN		CAN, CO	CAN, CO	CAN	
Galactose => Glucose-1P							CA				
Sucrose => D-Fructose-6P => G3P				CAN							
Trehalose => Trehalose-6P => D-glucose-6P		CAN	CN	CAN	CAN				CN		CAN
Glycolysis EMP complete (9-10 reactions)						CA, CO		CAN, CO		CAN	
PPP complete (8-9 reactions)							CN		CAN		CA
PPP oxidative phase		CAN, CO			CAN, CO	CAN, CO			CAN		CA
PPP non-oxidative phase (5-6 reactions)	AA, CO		AA, CAN	CAN			AAN, CN, CO	CN, CO	CAN, CO	AAN, CAN, CO	CAN
PTS Phosphotransferase system	CAN, CO	CAN, CO	CAN, CO	CAN, CO	CAN, CO	CAN, CO	CAN, CO	CAN, CO		CAN, CO	CAN, CO
Gluconeogenesis		AAN	AAN, CAN, CO			AAN, CAN		AAN, CA		AAN	AAN, CAN
G3P => glycerol-3P	AAN, CO	CAN	CA, CO	AAN, CAN, CO	CN	AAN, CAN	AAN	AAN, CA	CAN, CO	AAN	
Glycerol => G3P	CN					CA, CO				CAN	
Pyruvate <=> Lactate (NADH)			CP	CO	CP					CA, CN	CP
D-Ribose => ribose-5P	CA	CO	CA						CA	AAN, CAN, CO	CAN
Xylose => Xylulose-5P							CAN				
Xylulose shunt to acetate	AA, CO	CAN, CO	CN		CAN, CO	AA, CAN, CO		AAN, CAN, CO	CN, CO		CAN, CO
Xylulose shunt to acetyl-CoA			CN						CN		AAN, CAN
Pyruvate oxidation PDH		CAN	AN, CN, CO	AAN, CAN			CAN, CO		CAN, CO	CAN, CO	CAN, CO
Pyruvate oxidation PFOR					CAN	CAN	AAN, CA	AA, CN	AAN		
Ethanol from Pyruvate => Acetaldehyde						CAN					
Ethanol from Acetaldehyde	CO	CAN, CO	CN	CAN	CO	CAN	CN, CO	CN, CO			CAN

CO: complete, AA: autotrophic aerobic, AN: autotrophic anaerobic, CA: customized aerobic, CN: customized anaerobic, CAN: customized aerobic and anaerobic, AAN: autotrophic aerobic and anaerobic, CP: carbon propanoate

Several sulfur-oxidizing strategies were identified, including the oxidation of thiosulfate by the sulfur-oxidizing (SOX) complex, found in PO and both TH88 and TH94 MAGs, as well as the sulfite oxidation by sulfite-oxidizing enzymes (SOEs) complex, linked to sulfite detoxification from DSR, or after sulfite liberation from organo-sulfur molecules [76], identified only in TH88 MAG (Fig. 3a). The sulfide produced by SRB may be oxidized to elemental sulfur by flavocytochrome *c*-sulfide dehydrogenase (FccAB) (only *fcc*A gene identified in both THs), with electrons being transferred to cytochrome c [76, 85] or be oxidized to polysulfide by sulfide: quinone oxidoreductase (Sqr) [76, 86] (Fig. 3a; Table 2). The Sqr is a membrane-associated single-subunit flavoprotein that in action generate polysulfides, which may be oxidized to sulfite by the dissimilatory sulfite reductase complex (DSRC(AB)EFH/MKJOP) [34, 76], identified in TH88 and SU MAGs (Fig. 3a). The sulfite may be further oxidized to adenylyl sulfate (APS) by the AprAB-QmoABC complex and finally, to sulfate by the sulfate activating enzyme, sulfate adenylyl transferase (Sat), generating ATP [76, 81] (Fig. 3a, dashed lines, Table 2). Interestingly, the flux profile showed most sulfur oxidation routes in the two MAGs, TH88 and DE, which presented flux through DSR in both directions (Table 3). The co-occurrence of sulfate respiration, and the reverse reactions of sulfate oxidation by DSR have been previously described [83, 87], as well as the co-occurrence of multiple sulfur oxidation pathways [88]. The co-occurrence of such strategies is especially important to thrive in habitats where strong gradients of oxygen and sulfide exist, such as mangroves [14, 77]. A previous study showed that mangroves could mitigate sulfide pollution through L-cysteine biosynthesis from L-serine and hydrogen sulfide [34]. All MAGs except SU showed gene content and flux profile for L-cysteine or L-homocysteine biosynthesis using sulfide (Fig. 3a; Tables 2 and 3).

We also identified several sulfur metabolism-associated components related to fluctuating and highly reduced conditions. Dimethyl sulfoxide (DMSO) can act as an alternate electron acceptor to support anaerobic respiration and two of three subunits of the DMSO reductase complex (DmsABC) [89, 90] were observed in DE (Table 2). However, flux through DMSO reduction to dimethyl sulfide was predicted for the Pseudomonadales MAGs PS82 and PO (Table 3). The heterodisulfide reductase (Hdr)-like complex, involved in DMSO respiration, aerobic thiosulfate oxidation and organic sulfur transformation [89], was identified in TH94, DE, PY and ZI (Fig. 3d). The *cbb3*-type cytochrome *c* oxidase, encoded by the *cco*NOQP operon, is involved in microaerobic SOB adaptation to low oxygen concentrations [88] and was present in most Gammaproteobacteria MAGs (Fig. 3c). Additional genes involved in sulfide and sulfite oxidation (*SUOX*, *sir*) [91], and thiosulfate respiration (*TST, psr*A, *ttr*AB, *tsd*A) [76] were also found in most MAGs, except ZI (Fig. 3a; Table 2). All Gammaproteobacteria MAGs showed the sulfate permease (*sul*P), which can transport or exchange a number of inorganic anions such as sulfate, nitrate, and chloride in prokaryotes [92], and sulfate/sulfite uptake and secretion fluxes were predicted in all MAGs (Tables 2 and 4). The *cys*PUWA operon, which couples the transport of sulfate with the hydrolysis of ATP [92], was identified in PY and AC, with flux predicted mostly using the anaerobic media (Table 3). Considering the sulfur-oxidizing strategies identified, the gene content and the flux profile indicate that PO, both THs, DE and SU could be working as SOB (Fig. 3a; Table 3). The sulfur metabolism flux profile is illustrated in Figure S1.

**Table 4:** Main compounds with uptake/secretion exchanges in different MAGs and/or conditions, highlighting the respective reactions with higher flux

Compound	Uptake by	Uptake media	Secreted by	Secreted media
Sulfate [e0]	all	AAN, CO, CAN	PO, TH88,TH94	CO, CAN
	AC, AL, PY, PO, PS82,SU: Sulfate=> Sulfite(cytochrome)		PO, TH88:SOX	
	DE, PS11,TH88,TH94,SU: SRB1		TH94:revDSR	
Sulfite [e0]	PS11,AL, PO, TH88,SU	CO, CAN	PS82,AL, PO, TH88,TH94,DE, SU, PY, AC	CO, CAN, AN
	AL, PO, PS11:Sulfite=> H2S(NADPH)		AC, AL, PO, PS82,SU, PY: Sulfate=> Sulfite(cytochrome)	
	PS11,TH88:Sulfite=> H2S(cytochrome)		DE: H2S=> Sulfite(cytochrome)	
	SU: Sulfite=> H2S(DsrC-L-Cysteine)		SU, TH94:H2S=> Sulfite(DsrC-disulfide-form)	
H2S [e0]	PS82,AL, PO, TH94,DE, SU, PY, ZI, AC	CO, CAN	PS11,PS82,AL, PO, TH88,TH94,DE, SU, AC	CO, CAN, AAN
	PY: Homocysteine + L-cysteine synthesis		AC, AL: Cysteine=> NH3+Pyruvate+H2S	
	ZI: L-cysteine synthesis		AL, PO: Sulfite=> H2S(NADP)	
	DE: H2S=> Sulfite(cytochrome)		PS11,DE, THs: Sulfite=> H2S(cytochrome)	
H2S2O3 [e0]	PS11,PS82,AL, PO, TH88,TH94,DE, SU, PY, AC	CO, CAN	PS11,TH88,SU	CO, AN
	PO, THs: SOX		PS11:SO3 + Mercaptopyruvate => Pyruvate + H2S2O3	
	AL, AC: Cysteine from H2S2O3+O-Acetyl-L-serine(trdrd)		TH88:Sulfate=> H2S2O3	
N2 [e0]	SU	CO	TH88	CO
	N2=> NH3		Denitrification	
NH3 [e0]	all	AAN	all	CO, CAN
	AC, DE, PY, PS11,TH94: Nitrification1		AC, DE, SU, ZI: L-Glutamine=> NH3+L-Glutamate	
	AL, PO, PS82:NH3=> Nitrite(menaquinone)		AL, DE, PO, PS11,PS82:L-Glutamate=> NH3+2-Oxoglutarate	
	DE, AC, PY, PS11,SU, TH88,TH94,ZI: NH3=> Nitrite(ubiquinone)		PS82,TH88,TH94:Nitrite=> NH3(NADH, NADPH)	
			PY: Aminoethanol=> NH3+Acetaldehyde	
NO [e0]	PS82,TH94,DE, SU, PY, ZI, AC	CO, CAN	PS82,DE, SU, PO, TH94,ZI	CO, CAN, AAN
	PS82,PO, TH94:NO=> Nitrate		Nitrite=> NO	
	AC, DE, PY, SU, TH94,ZI: Denitrification			
Nitrite [e0]	PS11,PS82,AL, PO, TH88,TH94,DE, PY, AC	CAN	PS11,PS82,AL, TH88,PY, AC	AAN
	AC, TH88,TH94:Nitrite=> NH3(NADH)		AC, PY, PS11,TH88:NH3=> Nitrite(Ubiquinone)	
	DE: Nitrite=> NH3(Reducedferredoxin)		AL, PS82:NH3=> Nitrite(menaquinone)	
	DE, PY: Nitrite=> NH3(Ubiquinol)			
	PO, TH94:Nitrite=> NO(Cytochrome)			
Formate [e0]	PS11,DE, SU, ZI	CO	AL, PO, PY	CO, CN
	DE, ZI: WLP Methyl branch		AC, AL, PY, PO, PS11: 10-Formyl-THF=> Formate	
	SU: Formate => CO2 (cytochrome)			
Acetaldehyde [e0]	PS11,PS82,PO, TH88,TH94,DE, SU, ZI, PY	CO	ZI	CAN
	Ethanol from Acetaldehyde (all -PY, ZI)		L-Threonine => Glycine + Acetaldehyde	
			L-Lactate => Formate + Acetaldehyde	
Acetate [e0]	PS11,PO, PY, AC	CAN	all	CO, CAN
	PO, AC, PY: Acetate oxidation		AC, AL, PY, PS11,PS82,TH88,TH94,SU: D-Xylulose-5P=> Acetate	
	PY, PS11:Acetate=> Acetyl-P=> D-fructose-6P		All -SU: L-Cysteine/Homocysteine from H2S and H2S2O3	
Acetoacetate [e0]	DE	CO	PY	CO
	Butanoate from Acetoacetate (DE)		Succinyl-CoA + Acetoacetate <= Succinate + Acetoacetyl-CoA	
Ethanol [e0]	PY	CO	PS11,PS82,AL, PO, TH88,TH94,DE, SU, AC	CO, CAN
	Ethanol utilization		Ethanol from Acetaldehyde (all -PY, ZI)	
L-Aspartate [e0]	PS11,PS82,AL, PO, TH88,TH94,DE, PY, AC	CO, CAN	PS82,PO, PY	CO
Glycine [e0]	PS82,PY, SU, ZI	CO, CAN	PY	CA
L-Glutamate [e0]	PS11,PS82,AL, PO, TH88,TH94,DE, PY, AC	CO, CAN	AC	CA
L-Proline [e0]	PS11,PS82,PO, TH88,SU, ZI, AC	CO, CAN	PO, TH88	CO
Myristic acid [e0] (Tetradecanoate)	PS82	CO	PS82,AL	CA
D-Fructose [e0]	PS82,TH88, DE	CO, CAN	PS82,PO, TH88,DE	CO, CAN, AAN

### Nitrogen metabolism

The alternating aerobic and anaerobic conditions caused by tidal flushing in mangroves favor the co-occurrence of nitrification, denitrification and anaerobic ammonium oxidation [93, 94]; however, the high salinity and frequent anoxic conditions in mangroves decrease the rate of nitrification and retain nitrogen in reduced form, which may be assimilated especially by denitrifying bacteria [36, 79]. The genes from the first nitrification reaction were identified in most Pseudomonadales MAGs, while the second and third reactions were identified only in AC (Fig. 3b, blue arrows). Interestingly, besides lacking the genes according to the KEGG reconstruction, DE showed predicted flux through the nitrification pathway in the FBA using autotrophic media (Table 3).

The Gammaproteobacteria showed a higher abundance of nitrogen metabolism-related genes, but the pathways were scattered and mostly incomplete. Only TH94 showed the complete gene set required for nitrate reduction by dissimilatory nitrate reduction (DNR) (Fig. 3b, red arrows). However, the flux profile predicted DNR activity for most Gammaproteobacteria MAGs (Table 3), which also showed the *nir*BD genes, required for the second DNR step, reducing nitrite to ammonia (Fig. 3b). TH94 also showed the almost complete denitrification pathway, except by the last reaction, converting nitrous oxide to gaseous nitrogen, whose *nos*Z gene, coding for nitrous oxide reductase, was identified only in TH88 (Fig. 3b, green arrows). Consistently, TH94 showed predicted fluxes through DNR and denitrification until nitrous oxide, and only TH88 showed flux from nitrous oxide to gaseous nitrogen (Table 3). Similarly, besides TH94, fluxes through the second and third denitrification reactions were also observed for DE, SU and ZI (Tables 2 and 3). We also highlight the presence of the membrane-bound periplasmic *fdo*GHI operon, which codes for the FDH-O isoenzyme complex, typically involved in formate oxidation coupled to nitrate or nitrite reduction [95], which positive flux was observed for PS11, PO, TH94 and DE, indicating that formate oxidation may be coupled to nitrite reduction to ammonia by the presence of *nir*B and *nir*D genes encoded in *nir* operon (Fig. 3b; Table 3).

The genes required for nitrogen fixation were identified in TH88 and DE (Fig. 3b), which also showed predicted flux (Table 3). Genes involved in organic nitrogen cycling, on the other hand, were broadly distributed. A diverse set of genes working in the interconversion between glutamate and ammonia was observed in all MAGs, in agreement with the flux profile. The Pseudomonadales MAGs (except AL) showed the gene set for urea uptake (*urt*ABCDE) and hydrolysis to ammonia (*ure*ABC*)* (Fig. 3b), which may be linked to arginine biosynthesis, a urea-producing process, or glutamate/glutamine biosynthesis, which require ammonia. Fluxes for urea hydrolysis to ammonia were observed only for most Gammaproteobacteria MAGs (Table 3). We also highlight the NRT/*nar*K and *nrt*ABC extracellular nitrate/nitrite transporters, and the nitrilase enzyme (Fig. 3b), usually found in plant-associated bacteria, involved in detoxification, nutrient assimilation and modulation of plant development and physiology [96]. Further, all Gammaproteobacteria presented the complete Molybdenum cofactor biosynthesis module (M00880), which is involved in nitrate reduction in microaerophilic or anaerobic environments [95].

The coupled activity of SOB and nitrate-reducing bacteria was already observed in mangrove sediments [11, 97]. Both Thiohalomonadales MAGs, PO, and SU showed several genes and complexes potentially involved in sulfate/sulfite reduction, and sulfur oxidation coupled to nitrate/nitrite reduction, suggesting their possible role as S-driven denitrifiers [11] (Figs. 2 and 3; Table 3). TH94 showed the gene set for mixotrophic denitrification, a growth strategy in which heterotrophic denitrification and sulfur-based autotrophic denitrification may occur simultaneously [98–100]. TH94 showed the genes required for thiosulfate oxidation (*sox*), sulfite oxidation (*dsr*, *apr*, *qmo*) and sulfide oxidation (*sqr*) using nitrate reduction (*nap*AB) or nitrite reduction (*nir*S, *nir*BD) as its electron acceptors, and the genes involved in carbon fixation by Calvin cycle (with flux predicted), indicating that CO2 could be the sole carbon source [85, 98, 101]. The flux profile indicates the capability of TH94 to couple thiosulfate and sulfite oxidation to DNR and denitrification, as already described in mangrove sediments [11, 40, 102] (Fig. S1). The nitrogen metabolism flux profile is illustrated in Figure S2a.

### ATP synthesis

The five complexes of the oxidative phosphorylation pathway were mostly identified in the Gammaproteobacteria MAGs, which presented several electron transport systems and terminal respiratory cytochrome oxidases (Fig. 3c). Exchange fluxes through complexes II, III and IV were observed using ubiquinol and/or menaquinol as electron carriers (Table 3, Fig. S2b). We highlight the presence of genes of the V/A-type ATPase H+/Na+-transporting and cytochrome o-ubiquinol oxidase, identified only in DE (Fig. 3c; Table 2). Two membrane-bound ion-motive electron transport complexes and several ATPases based on ion gradients across the membrane, also called proton pumps, were broadly identified among the MAGs (Fig. 3d), with flux predicted. The RNF complex, a reversible membrane-bound ferredoxin-dependent oxidoreductase complex observed in many anaerobic bacteria [103, 104], plays a major role in electron flow and energy conservation in several metabolic pathways such as fermentation, nitrogen fixation, sulfate reduction and acetogenesis [105, 106], was identified in PS82, PO, both THs, DE and SU MAGs. The genes encoding the energy conserving NADH: ubiquinone oxidoreductase (NQR) complex, identified in all Pseudomonadales MAGs (Fig. 3d), mediates the electron transfer from NADH to quinone using sodium gradient, driving energy-dissipating processes such substrate uptake, ATP synthesis, cation-proton antiport, and iron uptake and regulation [104, 107]. We also identified the electron transfer flavoprotein (EtfAB), which participates in FAD and ferredoxin recycling [104], identified in most MAGs, except both Thiohalomonadales and PY. Several Na + translocating ATP synthases provide an advantage at haloalkaline conditions where extracellular Na + concentrations are high and thus contribute to the sodium motive force [104]. We also identified three cation-proton antiporters, which are not directly involved in ATP synthesis, but play important roles in pH, ion, and volume homeostasis by exchanging K+, Na + and Ca2 + for H + across the membrane in response to environmental conditions [108] (Fig. 3d). The NhaA Na+(Li+)/H + antiporter, along with its transcriptional regulator NhaR, were identified in both THs, while the almost complete Mnh/Mrp and Pha antiporter complexes were exclusive to TH94 and AL, respectively. These cation-proton antiporters work on K+:H + and Na+/H + exchange systems, acting on pH homeostasis, especially observed under alkaline conditions [108, 109]. We also identified the potassium-stimulated pyrophosphate-energized sodium pump encoding gene (*hpp*A) [28], present in most MAGs (Fig. 3d).

### Carbon metabolism

The carbon metabolism showed an interesting diversity of carbon sources and oxidation substrates. The most representative pathways were involved in glycolytic routes (glycolysis, pyruvate oxidation, starch metabolism and pentose phosphate pathway), carbon fixation and acidogenesis (acetate, butanoate and propanoate metabolism).

Several CAZy enzymes from the Glycoside Hydrolase family were identified, 34 Glycosidases (EC 3.2.1.-) and 28 Hexosyltransferases (EC 2.4.1.-) (Table S4), as previously observed in mangroves [24]. PY and ZI showed the highest richness of Glycoside hydrolases (19 and 10, respectively), and PY and AC showed the highest richness of Hexosyltransferases (14 each). Galactose degradation module was complete on DE, and the Trehalose biosynthesis module was complete in SU (Table S3). The trehalose-mediated resistance to osmotic stress is known to protect bacterial cells against several abiotic stresses [110]. Figure S3 presents the main polysaccharides, disaccharides, and monosaccharides (hexoses) degradation and conversion routes identified. The respective predicted fluxes identified are presented in Table 3. PY showed the required enzymes for starch, dextrin, maltose, trehalose, lactose and sucrose conversion to glucose, along with ribose and xylose by non-oxidative pentose phosphate pathway (PPP). Glycogen catabolism is an important source of energy (ATP) and reducing power (NADH2) under anaerobic conditions [111], and the complete glycogen degradation module was identified only in AC. However, most MAGs presented the glycogen phosphorylase (PYG) and the phosphoglucomutase (PGM), but the step involving the hexosyltransferases (*mal*Q/*jgt*) and the glycosidases (*glg*X/*pul*A) was absent, except in AC (Table 2).

**Fig. 4:**
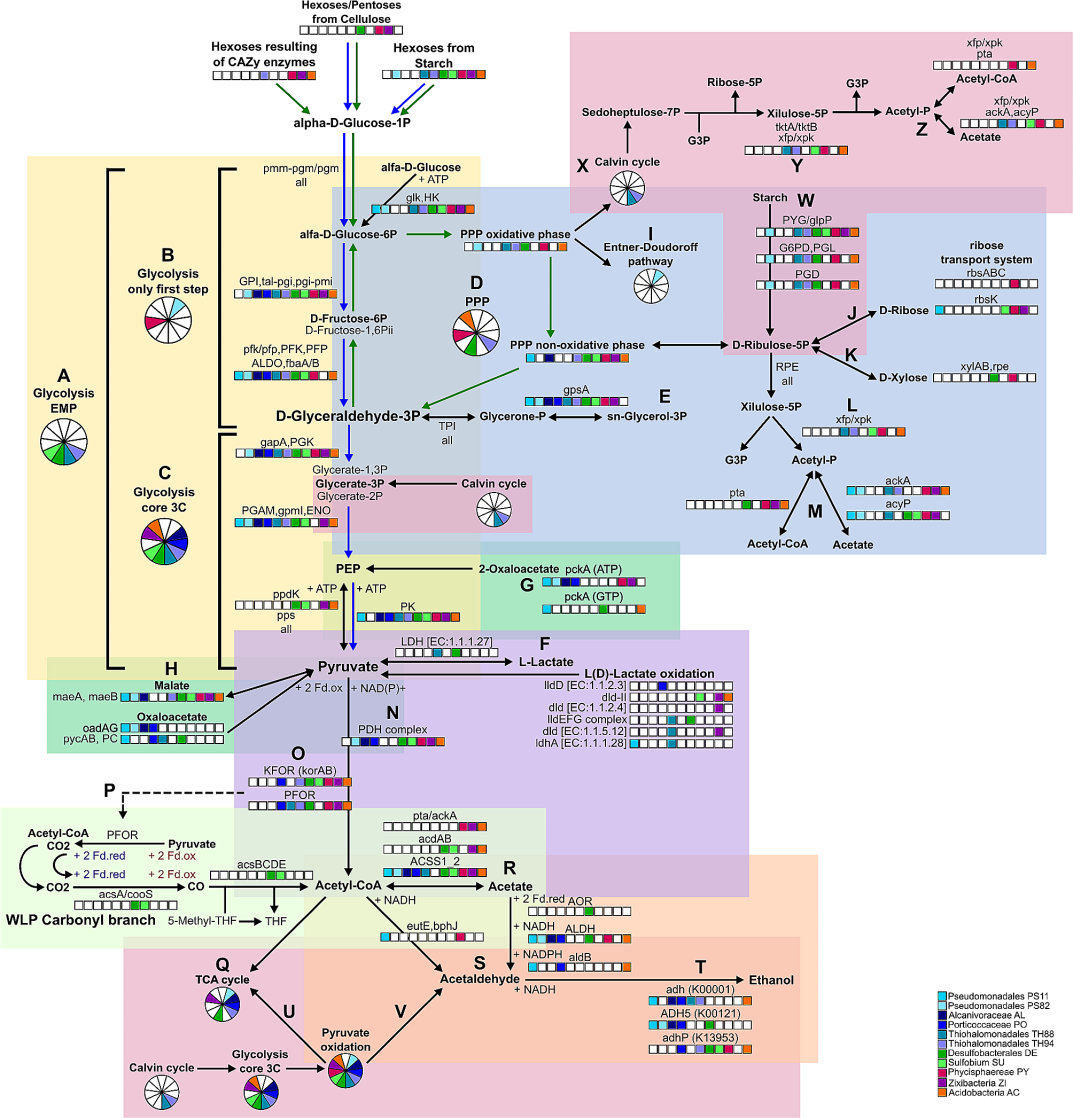
The glycolytic pathway and connections

The genes required for the complete glycolytic route, from complex carbohydrates breakdown to subsequent oxidation until the 3 C compounds via glycolysis were identified in PS82, TH88, TH94, DE, SU, ZI and AC (Fig. 4, blue arrows), with flux from maltose breakdown to pyruvate predicted for TH94, SU and ZI (Table 3). On the other hand, genes involved in starch and other polysaccharides breakdown by Glycoside Hydrolase (GH) family, followed by PPP were observed in TH88, PY and AC (Fig. 4, green arrows), with flux from galactose, maltose, fructose, and trehalose breakdown throughout pentose phosphate PPP predicted for DE, PY and AC (Table 3). Only both THs, DE and SU showed the complete module of the Embden-Meyerhof-Parnas (EMP) pathway (M00001), the most common type of glycolysis (Fig. 4a), while the remaining MAGs (except PS11) missed one step or presented a different enzyme than the KEGG module M00001 (Fig. 4b and c; Table 2). The complete PPP gene set was identified in TH88, DE, PY and AC (Fig. 4d). Flux throughout the EMP glycolysis was observed for TH94, SU and ZI, while flux throughout the PPP was observed for DE, PY and AC, indicating no co-occurrence of the two pathways (Table 3). In the first step of EMP glycolysis, glucose is phosphorylated to produce glucose-6P using ATP. However, in bacteria, phosphoenolpyruvate (PEP) can be the substrate for glucose phosphorylation via the phosphoenolpyruvate (PEP)-dependent phosphotransferase system (PTS) [112]. The PTS may be used as an energy-saving strategy, coupling the hexose phosphorylation to sugar catabolism and anabolic routes, especially glycogen biosynthesis [112]. The KEGG reconstruction captured only 10 PTS genes. The FBA analysis, on the other hand, predicted flux for PTS reactions using seven different phosphorylation substrates in most MAGs (Table 3, Table S5).

The anabolic gluconeogenesis pathway was incomplete according to KEGG reconstruction. However, we were able to identify flux from the first reaction (converting oxaloacetate to PEP) until the last reaction (generating D-fructose-6P) in AL, TH94 and AC, especially in the autotrophic media (Table 3). All MAGs except AC showed the genes required for glycerol-3P synthesis (*TPI* and *gps*A), which may enter Glycerophospholipid metabolism [113] (Fig. 4e). Most MAGs showed predicted flux from Glyceraldehyde-3P (G3P) to glycerol-3P, especially considering the autotrophic media, and PS11, TH94 and ZI showed flux through the conversion of glycerol to G3P (Table 3). The lactate dehydrogenase, the enzyme required for the interconversion between pyruvate and lactate, was present in TH88 and DE (Fig. 4f). Another six genes involved in lactate/pyruvate conversion were identified in PO, SU, ZI and AC (Fig. 4f), with flux predicted for PO and ZI (Table 3). We also highlight the anaplerotic recycle of tricarboxylic acid cycle (TCA) intermediaries to PEP (Fig. 4g) and pyruvate (Fig. 4h).

Only PS82 showed the genes required for the coupled PPP oxidative phase and Entner-Doudoroff pathway, known to be used by *Pseudomonas* for hexose catabolism to G3P and pyruvate [114] (Fig. 4i), but no flux was predicted. The PPP oxidative branch produces the essential reductant NADPH and D-Gluconate-6P. The non-oxidative branch produces Ribose-5P from glucose and can be interconverted into glycolytic intermediates such as G3P and fructose-6P, and D-Glucose-6P [112] (Fig. 4, green arrows). All MAGs showed the ribose-phosphate pyrophosphokinase [EC:2.7.6.1] encoding gene, whose flux was predicted. This enzyme catalyzes the conversion of Ribose-5P to 5-phospho-d-ribosyl-α-1-diphosphate (PRPP), a ubiquitous precursor to the biosynthesis of the amino acids histidine and tryptophan, the cofactors nicotinamide adenine dinucleotide (NAD) and NAD phosphate (NADP), and the purine and pyrimidine nucleotides, which are the building blocks for RNA, DNA and the energy carrier molecules (ATP, NAD and NADP) [112].

PS11, PY, ZI and AC presented the predicted flux and genes required to convert D-Ribose to G3P through non-oxidative PPP, but only PY presented the genes encoding for ribose transport system (*rbs*ABC) (Fig. 4j; Tables 2 and 3). The genes required for oxidation of D-Xylose to G3P and fructose-6P were identified only in DE and PY (Fig. 4k). As a major component of vegetal biomass, the conversion of xylose to economically valuable products have been investigated, but only a few microorganisms can metabolize xylose naturally [115]. The xylulose-5P/fructose-6P phosphoketolase (xpf/xpk) [EC:4.1.2.9, 4.1.2.22], identified in both THs, SU, PY and AC (Fig. 4l), may potentially couple the non-oxidative PPP to the heterotrophic acetogenesis by converting xylulose-5P/fructose-6P to Acetyl-P and G3P/D-Erythrose-4P (xylulose shunt) [116]. The resulting acetyl-P may be converted to acetate by acetate kinase (ackA) [EC:2.7.2.1] or acylphosphatase (acyP) [EC:3.6.1.7], or be converted to acetyl-CoA by phosphate acetyltransferase (pta) [EC:2.3.1.8] [115] (Fig. 4m). Flux through the xylulose shunt to acetate was predicted in several MAGs, while flux through the xylulose shunt to acetyl-CoA was predicted only for AL, PY and AC (Tables 2 and 3).

Only PS11 presented neither flux nor any gene involved in pyruvate oxidation to acetyl-CoA (Fig. 4n and o). The encoding genes for pyruvate dehydrogenase (PDH), pyruvate: ferredoxin oxidoreductase (PFOR) and 2-oxoglutarate ferredoxin oxidoreductase (KFOR) complexes were broadly distributed, but PS82 and AL presented only the PDH complex (Fig. 4n), which couples the oxidative decarboxylation of pyruvate to the reduction of NAD+, mostly among aerobes [117]. PFOR catalyzes the oxidative decarboxylation of pyruvate to acetyl-CoA and CO2 using a low-potential reductant (ferredoxin or flavodoxin), mostly among anaerobes, especially under reduced conditions [117]. PS82, AL and PO presented flux only through the PDH complex, while both THs and SU presented flux only through PFOR complex (Table 3). Flux through both mechanisms was predicted in DE and PY (Table 3). The CO2 and the two reduced ferredoxin produced by PFOR in the oxidative direction may couple the autotrophic Wood-Ljungdahl pathway (WLP) to glycolysis [117] (Fig. 4p). In anaerobic organisms several electron carriers, such as cytochromes, menaquinone, ferredoxin, and flavoprotein generate electrochemical ion-gradient to synthesize ATP. The reduced ferredoxin is used as an electron donor in anaerobic metabolism to drive several kinds of redox reactions including CO2 reduction [118].

The acetyl-CoA resulting from pyruvate oxidation may follow four main catabolic routes: enter the oxidative TCA cycle, be converted to VFAs, especially acetate, be directly converted to acetaldehyde, or produce ethanol (Fig. 4q, r, s and t, respectively). No MAG showed the genes for the major pathway for ethanol production through direct decarboxylation of pyruvate to acetaldehyde; however, flux through this reaction was predicted for TH94 (Table 3). The FBA predicted ethanol production from acetaldehyde and secretion for most MAGs (except PY and ZI) (Tables 3 and 4). All Pseudomonadales, DE, PY and AC showed the genes of the oxidative pathway of ethanol production through the intermediates acetyl-CoA and acetate [119] (Fig. 4r, s and t), but no flux was observed.

### Carbon fixation through the Calvin-Benson-Bassham cycle

The Calvin-Benson-Bassham (CBB) is one of the most common carbon fixation pathways, by which the phosphoribulokinase enzyme (PRK) and the ribulose bisphosphate carboxylase/oxygenase (Rubisco), encoded by *rbc*LS genes assimilate CO2 to generate 3-phosphoglycerate [116, 120]. The autotrophic generation of biomass by Rubisco-catalyzed CO2 fixation in chemolithoautotrophics has been investigated for biotechnological purposes, because in naturally occurring bacteria, especially among Gammaproteobacteria class, the CBB may be coupled to glycolysis/gluconeogenesis, TCA cycle, PPP and glyoxylate cycle [116, 121]. Both THs presented most CBB genes from the photosynthetic module M00165 (Fig. S4), but the cycle was incomplete. Both THs presented the unique CBB genes *rbc*LS and phosphoribulokinase (Prk) (Table 2), with flux predicted for TH94 in aerobic and anaerobic autotrophic media, as also observed for PO (Table 5). Both THs missed the sedoheptulose-1,7-bisphosphatase enzyme [EC 3.1.3.37] from the M00165 module, but TH94 presented, instead, the bifunctional fructose-1,6-bisphosphatase I [EC:3.1.3.11] (FBP) [116]. The Rubisco of some chemolithoautotrophics, especially among Gammaproteobacteria, may also fix O2 to produce 2-phosphoglycolate, which may be oxidized to glyoxylate by phosphoglycolate phosphatase (PGP), and glycolate dehydrogenase [116], which genes required, *ghp* and *glc*DEF, respectively, were identified in PO and both THs (Fig. S4b), with flux predicted for the last reaction generating glyoxylate (Table 3).

Three phosphoglycerate mutases (one EC:5.4.2.11 and two 5.4.2.12) identified among all the MAGs compete with phosphoglycerate kinase and regulate the carbon flux, which may direct the glycerate-3P generated from CO2 in two ways: by the TCA cycle for biomass synthesis [116, 120] (Fig. 4u; Table 2), or to ethanol fermentation by coupling CBB with glycolysis [122] (Fig. 4v). Both THs showed the genes required to couple CBB to glycolysis and ethanol production via glycerate-3P [116], and flux through the shunt to ethanol from CBB was predicted for PO and TH94 (Table 5). The shunt for ethanol production from CBB has been investigated for bioengineering purposes to reduce the undesirable competing synthesis of glycerol, improving the ethanol production [122], as previously observed for a *Clostridium* isolated from a mangrove sediment in Thailand [123]. Both THs also presented the genes required for generating CBB intermediates from complex carbohydrates (starch) degradation through the PPP oxidative phase (Fig. 4w), a useful route to provide NADH/NADPH in a low-energy scenario [116]. Another route to avoid carbon loss is the generation of acetyl-CoA from acetyl-P via transketolase (TKT) and phosphoketolase (PKET) [116], whose genes (*tkt*A and *xfp*, respectively) were identified in both THs, SU, PY and AC (Fig. 4y), with flux predicted for acetyl-CoA and acetate synthesis in TH94, SU, PY and AC (Fig. 4z; Table 5). The energy source may come from light in photosynthetic microorganisms as well as from sulfur/sulfide and molecular hydrogen in non-photosynthetic microorganisms [116]. We observed components of SRB and SOB in both THs (Fig. 3a), suggesting a possible coupling between the CBB and sulfur metabolism in the Thiohalomonadales MAGs, especially TH94 (Table 3).

**Table 5:** FBA profile showing the predicted flux through the main carbon fixation and VFAs pathways

	PS11	PS82	AL	PO	TH88	TH94	DE	SU	PY	ZI	AC
Calvin PRK/rbcLS				AAN		AAN					
Calvin cycle (8-11 reactions)	CAN			AAN		AAN	CAN	CN	AA, CA		
Glycolate => Glyoxylate		CN	AN	AAN, CO	AAN, CN	CAN, CO	AAN	CAN			CO
shunt to ethanol from CBB				YES		YES					
Sedoheptulose-7P=> Xylulose-5P=> Acetyl-P=> Acetate						CAN, CO		AAN, CAN, CO	CN, CO		CAN, CO
Sedoheptulose-7P=> Xylulose-5P=> Acetyl-P=> Acetyl-CoA								AAN, CAN, CO	CN		AAN, CAN
oxTCA complete		CA	CAN, CO	CA, CO			CO		CAN, CO		
roTCA most reactions		AAN, CN	AAN		AAN, CN, CO	AN, CA					
rTCA most reactions			AA		AAN						
CO2 uptake	AAN, CAN	AAN	AAN	AAN	AAN	AAN	AAN	AAN	AAN	AAN	AAN
CO2 => H2CO3	AAN, CAN,	AAN, CAN, CO		AAN	AAN, CAN, CO,	AAN, CA,	AA, CAN		AAN, CAN, CO,	AAN, CAN, CO,	AAN
rTCA: PEP + CO2 => Oxaloacetate		AAN, CAN, CO			CAN, CO	AAN, CA	AN		AAN, CAN	AAN, CAN, CO	
rTCA: Acetyl-CoA + CO2 => Pyruvate (NADH)	AAN, CN	AAN, CO	AA, CA	CAN, CO			AAN, CA	AA	AAN, CA	AAN	AAN
rTCA: Pyruvate + H2CO3 => Oxaloacetate	AAN, CAN			AAN	CP		AA, CAN	CAN			AAN
Fumarate + Menaquinol => Succinate (roTCA, rTCA)	AA, AN, CA, CN,	AA, AN, CN	AA, AN, CN	AA, AN, CN	AA, AN, CN, CO	CA, CP	AA, AN, CA	CA, CN, CO			
Succinate => Succinyl-CoA (roTCA, rTCA)	AA, AN, CA, CN, CO	AA, AN	AA, CN	AA, AN	AA, AN, CN, CO	AA, AN, CA, CN, CO	AA, AN, CA			AA, AN, CA, CN	AA, AN, CA, CN, CO,
Succinyl-CoA => 2-Oxoglutarate			AA, AN		AA, AN						
CO2 + 2-Oxoglutarate => Isocitrate (rTCA/roTCA)			AA, AN								
Acetyl-CoA+CO2 => Pyruvate (NADH)	AA, AN, CN	AA, AN, CO	AA, CA	CA, CN, CO			AA, AN, CA	AA, AN	AA, AN, CA	AA, AN	AA, AN
WLP Methyl branch (5 reactions)							AAN, CAN, CO	AAN, CA, CO		CAN, CO	
WLP CO => CO2 (rxn40505_c0)					AAN, CP			AA, CP			
WLP reductive methyl branch							AAN, CO	AAN, CA, CO		CAN, CO	
WLP oxidative methyl branch	CAN	CAN	CA, CO	CAN, CO					CN	AAN	
Isocitrate => Glyoxalate + Succinate	AA	CO	AAN, CAN	CAN	AAN, CAN	AA, CAN, CO					AAN, CAN, CO
Acetyl-CoA + Glyoxalate => Malate	CA		AAN, CAN, CO	AAN, CAN, CO	AAN, CO	CAN					AAN, CAN
Malate => Pyruvate (NAD/NADP)		CAN	CN					CA	CAN		
GCS (rxn06377_c0, rxn06600_c0, rxn06493_c0)		AAN					AAN		AAN, CAN	CAN	CAN, CO
Acetate + ATP => Acetyl-CoA (1 reaction)	AAN, CAN	AA	AAN	AAN, CA	CN	AA	AAN	AAN, CA			
ackA(+)/pta(-) (oxidation)									CAN	AAN, CN	AAN, CA
Acetyl-CoA => Acetate + ATP (1 reaction)							CAN, CO	CN, CO	CO	CAN, CO	CN, CO
Acetyl-P => Acetyl-CoA			CN						CAN	AAN, CN	AAN, CAN
Acetyl-P => Acetate	AA, CO	CAN, CO	CN		CAN, CO	AA, CAN, CO		AAN, CAN, CO	CN, CO		CAN, CO
Acetate => gluconeogenesis			yes					yes		yes	yes
Acetate => Butyrate							CAN, CO				
Acetate + Homocysteine (H2S)	AAN, CAN, CO	AAN, CAN, CO	AAN, CAN, CO	AAN, CAN, CO		CAN	AN, CN		AA, CN	CAN	AAN
Acetate + Homocysteine (H2SO3)		CAN, CO		AA, CAN		AAN, CAN, CO	CAN				CA
Acetate + L-cysteine (H2S)	AAN, CAN	AAN, CAN, CO	AAN, CAN, CO	CA, AN, CN	AAN, CAN, CO	AAN, CAN	AAN, CAN		AAN, CAN	CAN, CO	AA, CAN
Acetate + L-cysteine (H2SO3)	CN	CAN	CN	AA, CA			CAN		CA		CAN
Acetogenesis from sugars							CN	CN, CO	CO	CA, CN	
Acetate export by Na+ proton pump				CAN				CAN			CN, CO
Acetate export by H+ proton pump	CN, CO	CAN, CO	CAN, CO	CO	CAN, CO	CAN, CO	CAN, CO	CAN, CO	CN, CO	CAN, CO	CAN, CO
Succinyl-CoA => methylmalonyl-CoA => Propionyl-CoA			CP							AAN	AAN, CAN, CP
Propionyl-CoA => 2-Methylcitrate => Pyruvate + Succinate								CP	CP		
Propionate => Propionyl-CoA	CP	CP	CP	CP	CP	CP	CP	AN, CN, CP		CA	CP
Propionate + ATP => Propionyl-CoA		CP				CP	CP	AN, CN, CP		CA	
Acetyl-CoA + Propionate => Acetate + Propionyl-CoA	CP			CP							
Acetate + Butyryl-CoA => Acetyl-CoA + Butyrate							CA, CN, CO				
Acetoacetyl-CoA => Crotonyl-CoA => Butyryl-CoA	AAN, CA				AAN, CAN	AAN		AAN, CA			AN
Butyryl-CoA => 3-Hydroxyisobutyryl-CoA => Propionyl-CoA	CN										

### TCA cycle, reductive TCA, and connections

**Fig. 5:**
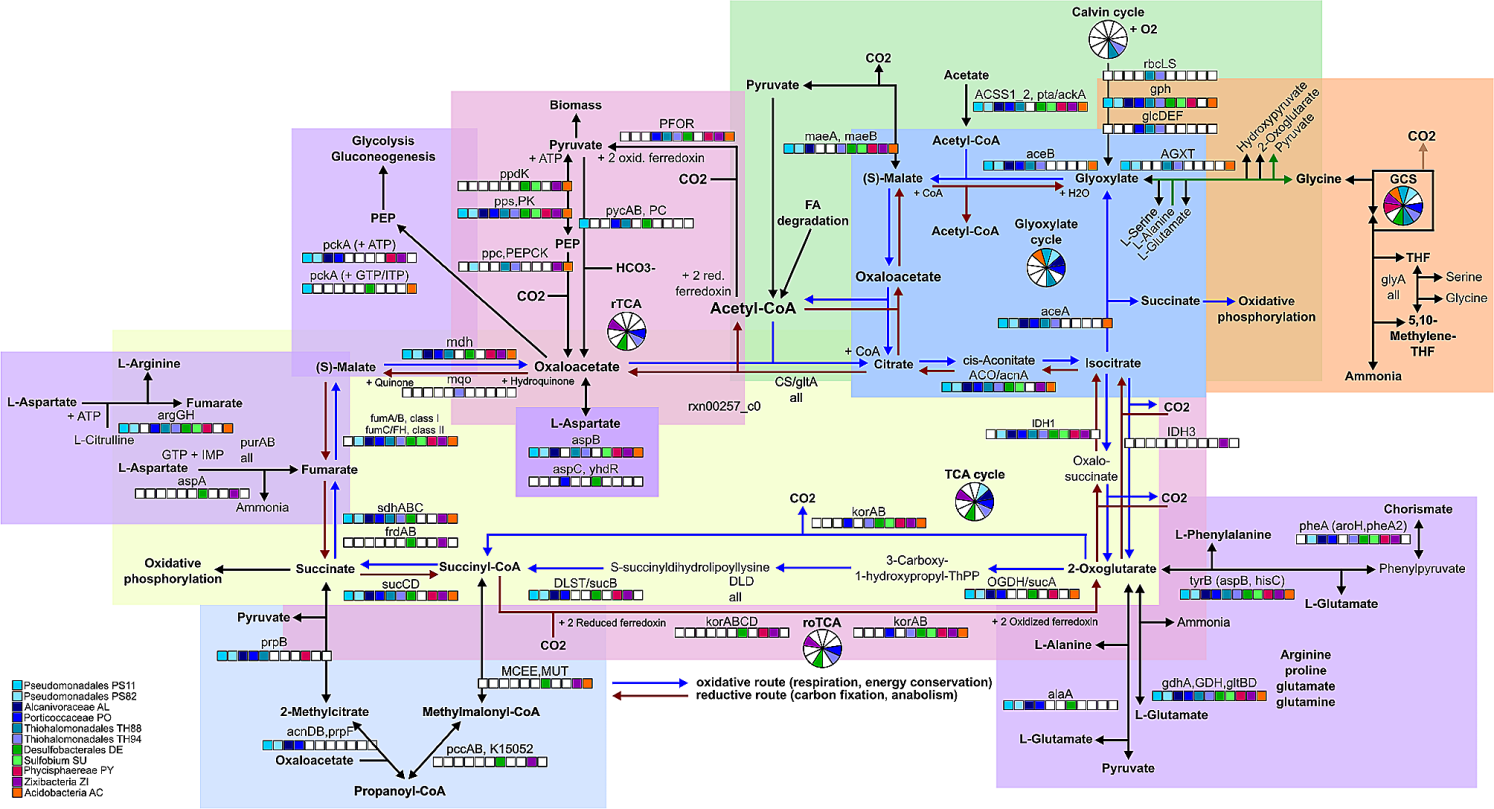
TCA cycle, variations and connections

The oxidative TCA (oxTCA) generates intermediaries and electrons for the respiratory chain and ATP synthesis (oxidative phosphorylation) in aerobes and anaerobes. In contrast, the reductive TCA cycle (rTCA) is a carbon fixation pathway used by many anaerobes, incorporating the acetyl group of acetyl-CoA into cell carbon and generating metabolic intermediates [119, 120, 124]. The oxTCA pathway was complete in most Gammaproteobacteria MAGs, DE and ZI (Fig. 5, yellow square, blue arrows), confirmed by the flux profile (Table 5). The same set of genes from the oxTCA can work reversely (roTCA), allowing bacteria to use the roTCA cycle to assimilate carbon when the CO2 partial pressure increases [124, 125]. The flux profile indicates both oxidative and reverse TCA capability (Table 5). Both rTCA and roTCA use 2-oxoglutarate: ferredoxin oxidoreductase (KFOR), encoded by *kor*AB, to convert succinyl-CoA to 2-oxoglutarate, and rTCA uses PFOR to synthesize pyruvate from acetyl-CoA using ferredoxin as electron donor and assimilating CO2 [117, 119, 124]. The required genes for the rTCA were identified in PO, DE and ZI (Fig. 5, yellow and pink squares, red arrows). The rTCA flux profile was incomplete, with both KFOR (*kor*AB) and PFOR complex acting in the oxidative direction (Table S5). The flux through the remaining rTCA reactions, on the other hand, was broadly observed, especially for AL and TH using the autotrophic media, as expected (Table 5). The reductive carboxylation of acetyl-CoA and CO2 to pyruvate by PFOR complex, the first step of the carbon fixation through rTCA (Fig. 5, pink square), is an energetically unfavorable reaction that requires a strong reduction potential and a strong electron donor as ferredoxins, along with high CO2 concentration [118], which is probably the reason why only oxidative flux was observed for this reaction. Interestingly, flux through the reductive direction of PDH complex, generating pyruvate from acetyl-CoA and CO2 using NADH, was broadly distributed, especially considering the FBA using the autotrophic media (Table 5). The acetyl-CoA required for pyruvate synthesis may be generated by the autotrophic WLP, whose complete set of genes was identified in DE and SU according to KEGG database, and flux predicted for the reductive methyl-branch in DE, SU and ZI (Table 5). The genes required to convert the autotrophically synthesized pyruvate to oxaloacetate (*pyc*AB, *PC*, *ppd*K, *pps/PK*, *ppc*/*PEPCK*), entering the rTCA cycle, or required to the anabolic route of pyruvate conversion to PEP *(ppd*K, *pps*) were broadly distributed (Fig. 5, pink square, Table 2), with flux predicted in most MAGs (Table 5). The rTCA cycle may be used in both directions to run anaplerotic reactions, generating metabolic intermediates for amino acid synthesis [119]. The oxaloacetate may be redirected to recycle PEP (*pck*A) or to aspartate biosynthesis (*asp*B, *asp*C, *yhd*R), and 2-oxoglutarate may be interconverted to pyruvate and glutamate, generating alanine and phenylalanine (Fig. 5, purple squares). Succinyl-CoA and succinate may be generated by the propanoyl-CoA synthesis and degradation routes (Fig. 5, light blue square). The numerous connections and the TCA cycle reversibility depending on the availability/concentration of organic versus inorganic carbon contributes to the flexibility required to survive in fluctuating environments as the microhabitats created by the tidal regime in mangrove sediments, where the configuration of autotrophy or heterotrophy is an ecological requirement [124, 125], responding to the porewater physical-chemical composition [3, 4, 8, 18, 40, 75].

The Glyoxylate cycle, one of the most representative pathways for the Pseudomonadales MAGs (Fig. 5, blue square), is a variation of the TCA, which converts acetyl-CoA to succinate for the synthesis of carbohydrates, bypassing the oxidative steps in the TCA cycle that loss carbon by producing CO2 [111]. Glyoxylate cycle activity is essential for microbial growth using acetate as a sole carbon source, and this pathway is reported to be up-regulated in the presence of acetate under anaerobic conditions [111]. The flux profile indicates that acetate oxidation coupled to the glyoxylate cycle may be active (Table 5). The glyoxylate bypass by the isocitrate lyase/malate synthase AceAB (*ace*AB) allows the accumulation of four-carbon precursors as succinate during growth on two-carbon substrates as acetate [111]. Glyoxylate cycle also plays a major role in oxidative stress and metabolic homeostasis [126]. The glyoxylate cycle may also be coupled to glycolysis by the interconversion of C3 carbon intermediates from glycolysis with C4 carbon intermediates from the glyoxylate cycle. This coupling mechanism occurs in the pyruvate-malate cycle, in which the malate is converted to pyruvate by the malic enzymes MaeA and MaeB. The pyruvate is then oxidized to acetyl-CoA, working in the balance of reducing power under anaerobic conditions [111]. The pyruvate-malate cycle coupled to the glyoxylate cycle was identified in most MAGs (Fig. 5, green and blue squares), with flux predicted (Table 5). We highlight the gene encoding the multifunctional glyoxylate transaminase (*AGXT*), identified in both PSs, both THs, and AC MAGs. This enzyme connects the glyoxylate metabolism to serine, alanine, glycine and pyruvate metabolism, as well as to the glycine cleavage pathway (GCS) [127] (Fig. 5, orange square).

The GCS, identified in most MAGs, catalyzes the cleavage of glycine to CO2, 5,10-methylenetetrahydrofolate (methylene-THF) and ammonia, with flux predicted for PS82, DE, PY, ZI and AC (Table 5). The GCS may also run in the reductive direction (reductive glycine pathway, rGCS) in many anaerobic microorganisms, working as a carbon fixation pathway, requiring ammonia and methylene-THF supply [128]. The autotrophic growth using the reductive GCS as the sole carbon fixation pathway was already reported [128], but the detailed role of each of its possible elements has not yet been fully described. The Methyl branch of WLP has been described as the first step of CO2 uptake in the autotrophic reductive glycine pathway, generating 5,10-methylene-THF [129].

### Acetogenesis and acetate oxidation

Acetate is a 2-carbon VFA which, together with propanoate (3-carbon) and butanoate (4-carbon), is a reduced intermediary generated from anaerobic oxidation of organic matter mostly in anoxic, energetically unfavorable environments, with a strong contribution to the global carbon cycle [119]. Acetate reduction and oxidation may be coupled with multiple metabolic pathways, working as a major intermediate product during the anaerobic decomposition of organic matter in a variety of environments, including hypersaline waters, paddy soils, and deep subsurface sediments [119, 130–133] and has been extensively investigated in anaerobic digestion bioreactors, organic waste treatment plants, and biogas production [134–140].

**Fig. 6:**
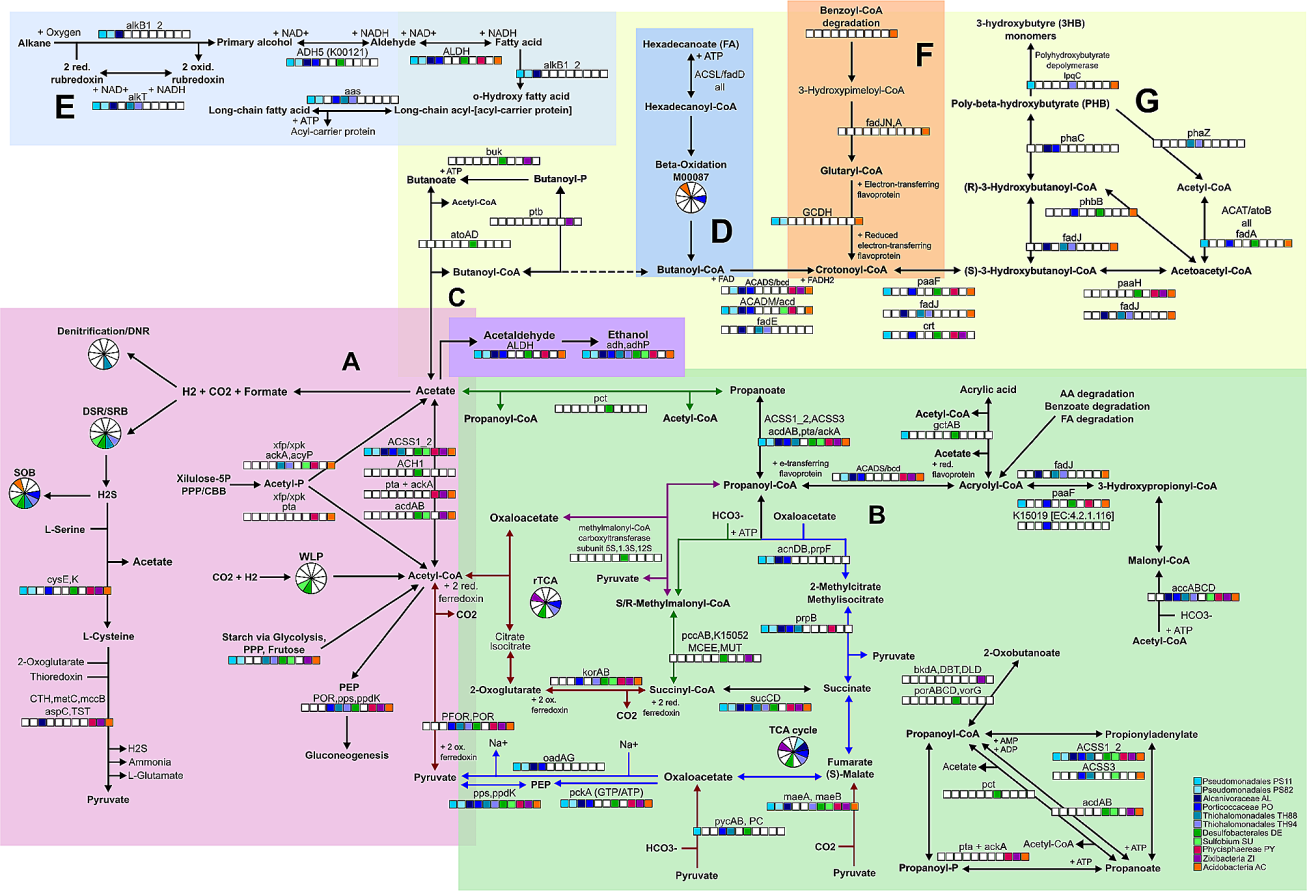
Volatile fatty acids production and oxidation, and fatty acid degradation: **A**: acetate, **B**: propanoate, **C**: butanoate, **D**: beta-oxidation, **E**: alkane degradation, **F**: benzoyl-CoA degradation

We identified 21 genes directly involved in acetate reduction and oxidation, one transcription factor and one acetate transporter, in addition to gene sets potentially involved in autotrophic and heterotrophic acetogenesis, acetate oxidation coupled to anabolic glycolytic routes [114–116, 130, 133], carbon fixation by reductive TCA cycle [119], VFAs production/recycling and ethanol production [119, 140], complete acetate oxidation by acetyl-CoA synthetase ACSS1_2 and oxidative WLP coupled with nitrogen and sulfur metabolism [101, 131, 141] and L-Cysteine biosynthesis [34] (Fig. 6a; Table 2). The FBA predicted flux for heterotrophic acetogenesis (DE, SU, PY, ZI, AC), acetate oxidation coupled to gluconeogenesis (AL, SU, ZI and AC), butanoate production and acetate as a byproduct of L-cysteine and homocysteine biosynthesis (Table 5).

Genes potentially involved in autotrophic and heterotrophic acetogenesis were mostly identified in the no-Gammaproteobacteria MAGs (DE, SU, PY, ZI, AC) (Table 2). Acetogens are anaerobic bacteria that may generate acetate and ATP autotrophically from the CO2 + H2 reduction to acetyl-CoA via WLP (homoacetogenesis) or heterotrophically using a variety of organic substrates as electron donors [119, 131, 135, 140]. The WLP complete module was identified in DE and SU. The phosphate acetyltransferase-acetate kinase pathway (M00579), identified in PY, ZI and AC, comprises the phosphotransacetylase (PTA) and the acetate kinase (Ack) and is the classical energy conservation step in autotrophic acetogens which assimilate CO2 through WLP [119, 133]. However, the WLP genes were absent in PY, ZI, and AC, and we could not predict flux through the entire WLP, but for most reactions of the WLP methyl-branch, from formate and tetrahydrofolate (THF) to 5-methyl-THF, consistently observed in DE and SU (Table 5). Further, the flux predicted through PTA/Ack in PY, ZI and AC was in the oxidative direction (Table 5). The flux through heterotrophic acetogenesis from sugar catabolism was predicted for DE, SU, PY, ZI and AC (Table 5). This route helps minimize the CO2 loss from sugar catabolism, a desirable capability for developing biotechnological biotransformation systems [135]. Since the oxidative direction was predicted for PTA/Ack, the acetate synthesis with ATP generation may be performed by the two subunits of ADP-forming acetate—CoA ligase (*acd*AB), identified in DE, SU, ZI and AC, which catalyzes the acetate formation and ATP synthesis from acetyl-CoA for anabolic acetate assimilation in bacteria and archaea [141, 142], or by the acetyl-CoA synthetase (ACSS1_2/ACS, EC:6.2.1.1) [140, 143], which was identified in all MAGs, except TH88 (Table 2).

Parallel to H2 consuming autotrophic acetogenesis, syntrophic acetate oxidizing bacteria (SAOB) oxidize acetate to CO2 and H2 typically through the reverse (oxidative) WLP, which donates the electrons from acetate to a terminal acceptor like sulfate or nitrate from a syntrophic partner [119, 140, 144]. The acetate may be converted to acetyl-CoA by the activity of ACSS1_2/ACS [140, 143], but also by the oxidative direction of PTA/Ack and *acd*AB. Both DE and SU presented several genes required for the SAOB activity, considering the most typical SAOB gene set [140, 141, 145] (Fig. 6a). However, only the carbon-monoxide and formate conversion to CO2 reactions from oxidative WLP showed flux for SU and TH88, both in the autotrophic media (Table 5). The acetate oxidation coupled to sulfate reduction is characteristic of SRB and SAOB in reduced conditions and sulfate-rich environments, where the production of sulfide inhibits the growth of methanogens [34, 101, 137]. Interestingly, besides both SRB and SAOB gene sets, SU also presented genes involved in sulfite and sulfide oxidation (Fig. 3a; Table 3). The flux through the complete WLP carbonyl branch was not observed. However, the gene set identified and the flux predicted for acetate oxidation and formate/CO conversion to CO2 in SU suggests that it may perform complete acetate oxidation coupled with sulfate respiration.

Acetogens and SAOB thrive under alkaline, highly reduced conditions, which are energetically unfavorable environments [137, 141], and additional energy may be provided by proton-mediated electrochemical gradients [141, 145]. Several H+/Na + proton pump ATPases were identified (Fig. 3d), including the RNF complex, characteristic of acetogens and SAOB [105, 106]. Flux was broadly predicted for acetate export using the H + proton pump, and using the Na + proton pump in SU, AC and PO (Table 5). Altogether, the metabolic features of acetogens, SAOB, and SRB provide tolerance to a wide range of pH, salinity, temperature, and high concentration of ammonia and suspended solids, successfully growing under conditions not tolerable by methanogens and methanotrophs [141, 143, 144, 146]. The absence of partial or complete methanogenesis or methane oxidation pathway among the eleven MAGs (gene content and FBA) makes us infer that at the time of sampling, the microhabitat conditions were antagonistic to methanogens or methanotrophs [78, 79, 141, 144], and the most abundant populations were those harboring the gene set for sulfate reduction and oxidation coupled to VFAs production, especially acetate, which is then oxidized, instead of being converted to methane, an interesting and desirable scenario considering the reduction of methane emissions [147].

### Propanoate and butanoate metabolism

Under anaerobic conditions, the complex organic matter may be directly oxidized to acetate and hydrogen, or generate reduced intermediaries such as propanoate, and butanoate, whose production and degradation are related to the inhibitory effect of acetate accumulation and thermodynamic equilibrium [119, 148, 149]. Most genes characteristic of syntrophic propanoate oxidizing bacteria (SPOB) were identified in DE, including the propanoate degradation via methylmalonyl-CoA pathway to succinyl-CoA, some key SPOB enzymes as the pyruvate carboxylase [EC:6.4.1.1] and the three-subunit transcarboxylase methylmalonyl-CoA carboxyltransferase [EC:2.1.3.1–5 S,1.3 S,12 S] (Fig. 6b; Table 2) [146, 149–152]. The three-subunit transcarboxylase is involved in the anaerobic Wood-Werkman cycle (WWC), which facilitates the anaerobic production of propanoate while maximizing energy capture coupled to CO2 fixation by pyruvate to oxaloacetate [150–152] (Fig. 6b, purple arrows). Additionally, several flavin-based electron bifurcations including EtfAB, heterodisulfide reductase HdrABC, and RNF complexes were identified, along with the ion-translocating pyrophosphatase HppA, the water-soluble divalent electron quinone oxidoreductase WrbA, and proton pump ATPases [149] (Fig. 3d). The succinyl-CoA produced from the propanoate degradation in SPOB may follow the reductive TCA cycle, in which specific genes (*kor*AB) were also present in DE (Fig. 6b, red arrows).

The genes required for propanoate production and oxidation via 2-methylcitrate were identified in the Pseudomonadales MAGs (Table 2). The FBA, on the other hand, showed an opposite profile, with propanoyl-CoA produced via methylmalonyl-CoA from succinyl-CoA in AL, ZI and AC, and propanoyl-CoA degradation to succinate and pyruvate via 2-Methylcitrate in SU and PY (Table 5). Flux through propanoate conversion to propanoyl-CoA was predicted for PS82, TH94, DE, SU and ZI (Table 5). Flux through acetate conversion to butanoate and acetyl-CoA was predicted for DE (Fig. 6c; Table 5). Flux throughout the acetoacetyl-CoA to butanoyl-Coa via crotonyl-CoA, generating 2-oxoglutarate, L-valine and pyruvate was predicted for PS11, both THs and DE, and the flux from butanoyl-CoA until propanoyl-CoA via (S)-3-Hydroxybutyryl-CoA, coupling butanoate and propanoate metabolism, was predicted for PS11 (Table 5).

### FBA: media comparison

Considering that the metabolic flux distribution of the community can be influenced by the interaction among the members and by the environmental conditions, which are highly dynamic in mangrove systems, the FBA based on the eleven GEMs investigated the interactive biochemical activities of the ETDI microbial community under fluctuating conditions of different media compositions, comparing the exchange fluxes and higher flow using the complete media, customized aerobic (CA), customized anaerobic (CN), autotrophic aerobic (AA) and autotrophic anaerobic (AN) media. The difference between the CA/CN and CA2/CN2 is that the last one does not present any amino acid in its composition. However, they were analyzed together in the flux profile. The composition of each media, attempting to simulate the fluctuating ecological conditions characteristic of coastal mangroves, is presented in Table S6. The occurrence and direction of the main reactions described in the flux profile are presented in Table S5.

The predicted maximum achievable flux through the biomass reaction of each GEM is presented in Table 6. The complete media predicted the higher objective values for all GEMs, with an average of 43.5. The customized aerobic media predicted higher growth rates of 6.4 for TH88 and TH94 GEMs. While testing for the best minimal media to adopt, we found that AL, PO, PY, ZI and AC grew better with the standard C-D-Lactate media, often used as minimal media, in comparison to all media compositions that we have tried, aside from the complete media, predicting an average of 2.26 objective value. The C-D-Glucose minimal media (refglumin), on the other hand, predicted an average of 0.09 objective value. The FBA comparison showed an average of 1744 reactions, from which around 404 were common to all GEMs. While the CN media showed the highest number of reactions, the complete media showed the highest number of compounds. The FBA analysis predicted 98 compounds presenting positive exchange flux, from which 27 were common between the complete and customized media, 53 were exclusive to CO media and 18 were exclusive to CA/CN media (Fig. S5, Table S7). We highlight sulfate, sulfite, sulfide, thiosulfate, ammonia, NO, and maltose among the common CO/CA/CN uptake compounds, trehalose, galactose, glycogen, sucrose, xylose, acetate, bicarbonate, aminoethanol, nitrate and nitrite among both customized media uptake exchange fluxes, and glutamate, formate, fumarate, acetaldehyde, ethanol and DMSO among the uptake compounds exclusive to CO media. On the other hand, a total of 55 compounds presented negative exchange flux, from which eight were common to CO, CA/CN and AA/AN media, eight were exclusive to CA/CN, and seven were exclusive to the AA/AN media (Table S7). We highlight sulfide, formate, D-fructose and succinate among the common secretion compounds, N2, fumarate, methyl sulfide, and acetoacetate among the compounds secreted only from CO media, acetaldehyde and glycogen(n-1) for CA/CN secreted compounds, and hydroxylamine and nitrite among the AA/AN secreted compounds (Table S7). A similar set of exchange compounds was already observed in mangrove sediments [13]. The community GEMs could predict 26 possible metabolic exchanges among the exchange flux compounds from each media (Table 4).

**Table 6:** Maximum achievable flux predicted through the biomass reaction of each GEM for the different media and FBA exchange fluxes comparison. CA/CA2*: aerobic customized media, CN/CN2*: anaerobic customized media, AA/AN: aerobic/anaerobic autotrophic media, LAC: C-D-Lactate standard media, refglumin: reference glucose minimal standard media. *CA2/CN2: indicate the media without amino acids

Media	PS11	PS82	AL	PO	TH88	TH94	DE	SU	PY	ZI	AC
Complete	43.51	45.76	25.95	34.53	42.77	47.3	55.99	51.64	39.72	41.5	50.33
CA	1.36	3.87	2.05	1.61	6.41	6.34	3.75	**2.31**	2.12	2.02	1.76
CA2	1.14	**5.9**	1.6	**3.1**	**7.5**	**6.5**	**7.6**	0.7	**4.1**	**3.7**	**6**
CN	0.8	2.94	0.26	0.87	3.94	1.4	3.75	2.14	2.02	2.02	0.33
CN2	**1.4**	3	7	0.7	6.4	3.7	7.4	1	7.6	1.4	7
AA	0.5	0.63	1.3	0.7	0.9	0.6	0.4	0.5	0.7	2	0.8
AN	0.01	0.26	0.32	0.33	0.21	0.3	1.1	0.1	0.28	1.97	0.24
LAC	0.53	2.56	**2.44**	2.51	0.96	2.97	2.58	1.23	3.28	3.4	2.43
refglumin	0.042	0.09	0.09	0.08	0.14	0.11	0.14	0.07	0.1	0.08	0.1

We observed a substantial difference between the flux profiles using the autotrophic media AA/AN and the flux profiles using the customized CA/CN and CO media. The models using the autotrophic media predicted uptake of CO2, ammonia and sulfate. Indeed, CO2 uptake was observed in all models, only using autotrophic media (except PS11, with CO2 uptake also for CA/CN). Flux through most reactions from rTCA was predicted, mostly using the autotrophic media (Table 5). However, the PFOR complex showed predicted flux only in the oxidative direction, and flux through pyruvate synthesis from acetyl-CoA and CO2 was predicted using NADH as the acceptor, instead of ferredoxin. Similarly, most reactions from the CBB showed predicted flux for both AA/AN media, including the rbcLS/PRK reaction (Table 5). Flux through bicarbonate synthesis from CO2 and subsequent oxaloacetate synthesis from pyruvate and bicarbonate were also broadly predicted in the autotrophic models (Table 5). The ammonia uptake was broadly distributed, but the higher positive fluxes were predicted mainly using both autotrophic media (Table 4). In both autotrophic media, ammonia was mostly converted to nitrite using ubiquinone and menaquinone, converted to L-glutamate using 2-oxoglutarate, and converted to hydroxylamine, the first nitrification reaction. In fact, the nitrification pathway was one of the pathways with flux predicted only using the autotrophic media (Table 3). Ammonia is also required for the GCS, which also showed higher predicted fluxes using the autotrophic media (Table 5). Flux through the complete DSR was predicted in both AA/AN media for TH88 and DE; however, flux through the sulfate reduction to APS, the DSR first step, and the sulfate reduction to sulfite using cytochrome c as electron acceptor were predicted for all models, mostly using AA/AN media (Table 3).

Among the compounds with higher secretion flux predicted, we highlight NO, resulting from the second denitrification reaction, with higher fluxes using the aerobic autotrophic media. Sulfide and sulfite, on the other hand, showed higher secretion flux using the anaerobic autotrophic media, with higher fluxes observed for sulfide resulting from sulfite reduction and sulfite resulting from sulfate reduction, both using cytochrome c as electron acceptor. The thiosulfate reduction from elemental sulfur and sulfite, with subsequent thiosulfate secretion, showed flux exclusively for the SU anaerobic autotrophic media. Hydroxylamine and nitrite resulting from the nitrification pathway and sulfite resulting from sulfate reduction were also among the compounds with higher secretion fluxes using autotrophic media, as described above.

Similar to the manual KEGG reconstruction, the flux profiles indicate the co-occurrence of SRB and SOB activity. Interestingly, flux through DSR was predicted in both reductive and oxidative directions, but SRB activity was mostly observed in the autotrophic media, while the oxidative direction was observed in CA, CN and CO media (Table 3). Other interesting pathways and routes with higher fluxes predicted using both autotrophic media were gluconeogenesis, the shunt to ethanol production from CBB, the propionyl-CoA synthesis via methylmalonyl-CoA, the butyryl-CoA synthesis from acetoacetyl-CoA and L-valine synthesis from butyryl-CoA generating pyruvate and 2-oxoglutarate (PS11, TH88, TH94, SU, AC) (Tables 3 and 5).

The major difference between the complete and both customized media was the exchange compounds since the CO media consisted of the compounds with respective transporters identified. The complete media predicted uptake flux for most amino acids, while no amino acid uptake flux was predicted in the customized media without amino acids CA2/CN2. The only amino acid with secretion flux in CA2/CN2 was glycine, only in PY, which was mainly produced from THF and glyoxylate (*gly*A and *AGXT* genes, respectively). The amino acid biosynthesis and degradation pathways identified by the KEGG reconstruction are presented in Fig. S6. The main pathways with flux predicted using the complete and/or the customized media were N fixation, DNR, reverse DSR, SOX, TCA cycle, complex carbohydrates degradation and glycolysis EMP, and ethanol production from acetaldehyde. The sulfur and nitrogen metabolism flux profile comparison between the complete and customized media are presented in Fig. S1 and Fig. S2. The complete PPP, ethanol production from direct oxidation of pyruvate to acetaldehyde, and aminoethanol uptake and utilization presented flux predicted only using the customized media. The models using the complete and customized media also showed several medium and long-chain fatty acid with predicted uptake fluxes, especially octadecanoate (PS11), hexadecanoate (PS82, AL, PO, TH94, DE, SU), tetradecanoate (PS82, AL), and decanoate (DE, SU, ZI). The complete set of reactions and steps of the FBA profile of the energy, glycolytic, carbon fix and VFAs pathways shown in Tables 3 and 5 are presented in Table S8, and the complete set of exchange compounds between MAGs shown in Table 4 is presented in Table S9.

Taken together, the exchange fluxes predicted between the MAGs considering the different media composition showed a wide range of individual metabolic capabilities and numerous inter-microbial metabolic interactions, in agreement with the two proposed scenarios (heterotrophic and autotrophic) in response to the tidal regime (Fig. 2).

### Living in community

Metabolic exchange within the microbial community is an essential process for developing and maintaining microbial ecosystems, allowing interconnections through the intermediary routes and syntrophy between metabolic partners [13, 14, 42]. Syntrophy or cross-feeding is the interaction between population or community members by sharing intermediates or final metabolites [41, 43]. During the syntrophic relationship establishment, some members may undergo minimization of cell complexity and specialization (streamlined genomes), becoming dependent on one or several metabolites produced by others, while other members keep the functions and present a highly diverse metabolism, capable of performing essential functions and becoming the so-called ‘keystone’ members, those that mostly sustain the syntrophic community [14, 41–43, 153]. The broad set of metabolic capabilities observed in ETDI MAGs cover most of the adaptations required for living in mangroves, and both THs, DE and SU shared several essential metabolic capabilities (Fig. S7), indicating their possible role as ‘keystone’ community members, that mostly sustain and drive the syntrophic community structure and functioning [14, 41–43, 153]. The SU MAG, classified as *Sulfobium*, belongs to Nitrospirota phylum, order Thermodesulfovibrionales, whose members are known as thermophilic strict anaerobes which grow by sulfate reduction to sulfide or sulfur disproportionation, coupled with oxidation of hydrogen or one-carbon compounds by the WLP [82–84, 87]. Members of this order may have both SRB and SOB gene sets, capable of sulfate respiration, but also the reverse reactions of sulfate reduction and sulfate oxidation [82, 83, 87]. Similarly, DE MAG, classified as belonging to the Desulfobacterales order, Desulfobacterota phylum, is also known as SRB, with members showing a highly diverse set of metabolic pathways [14, 105]. Interestingly, DE showed most metabolic features described for the new phylum Candidatus Cosmopoliota [40], described as a facultative mixotroph, including the co-occurrence of WLP and rTCA gene sets. The TH88 and TH 94 MAGs, classified as Thiohalomonadales order Gammaproteobacteria phylum, are known as facultatively anaerobic SOB, and frequently observed together with Desulfobacterales members among the most abundant populations in mangrove microbial communities [33, 34, 71]. Previous studies have shown members of this order as moderately halophilic chemolithoautotrophics capable of oxidizing thiosulfate and/or sulfide (electron donors) using nitrate as an electron acceptor, following the denitrification route until N2 as the final product [85]. Previous studies also describe carbon fixation by the CBB pathway and reductive TCA pathways [88]. Additionally, the multiple sets of electron donors and acceptors presented by these four MAGs allow the alternation of pathways yielding more energy in response to the environment [13, 14].

While these MAGs shared the major carbon and energy pathways, only TH94 presented the gene set required and flux predicted for simultaneous desulfurization and denitrification. Additionally, only DE showed evidence of acetate/butanoate conversion. SU showed several SAOB features, and both DE and SU presented genomic content and flux evidence to support their possible role as autotrophic acetogens under limiting conditions. The coupled sulfur and nitrogen metabolism and the evidence for syntrophic VFAs production and oxidation are essential to prevent inhibitory effects of high sulfide/nitrite concentration and acetate accumulation, respectively [119, 148, 149]. As previously described for keystone taxa from mangroves, TH94, DE and SU may support growth under fluctuating environmental conditions, potentially playing essential roles for the community stability and persistence [13, 14, 153, 154].

The other seven MAGs showed distinctive metabolic capabilities, although it was not possible to observe streamlining characteristics [13, 14, 42] in their genomes that could define them as specialists or keystones. The Pseudomonadales MAGs presented the highest number of lipid metabolism-related genes, especially those involved in fatty acid degradation. The beta-oxidation (M00087) was complete in PO and AC and only one gene was missing in the remaining Gammaproteobacteria (Fig. 6d). Most Pseudomonadales MAGs presented the genes required for aerobic alkane degradation to ω-hydroxy fatty acid (Fig. 6e). Alkanes are an abundant lipid in mangroves, originating from vegetation leaf wax [18], but are also associated with hydrocarbons [71]. It has been reported that native mangrove microbial communities may have the potential to degrade petroleum hydrocarbons, even in pristine mangroves [155]. Coupled with beta-oxidation, most genes required for the synthesis of poly-beta-hydroxybutyrate (PHB) were identified in PO, which showed the three reaction steps catalyzed by acetyl-CoA acetyltransferase (ACAT), acetoacetyl-CoA reductase and PHA synthase (PhaC) [156, 157]. The metabolic capability described for the Pseudomonadales MAGs seems to be more suitable for transient microaerobic/aerobic conditions, generating energy mostly from fatty acid degradation to acetyl-CoA, channeled through glyoxylate cycle, oxidative TCA and electron transport chain, with higher ATP yielding when oxygen is available. We also highlight the presence of genes required for ethanolamine synthesis from glycerol or glycerone-P in both PSs and AL [113].

The Acidobacteria MAG showed the complete anaerobic degradation of benzoyl-CoA ATP-dependent pathway (M00541) (Fig. 6f), potentially capable of deriving carbon from benzoate, as previously described for Acidobacteria [158]. However, the enzymes required to convert benzoate to benzoyl-CoA were missing in all MAGs, and no flux was detected. AC, PY and ZI shared several pathways, especially degradation of complex sugars and pentoses, carbon fixation reactions from different autotrophic pathways, as the PTA/Ack acetogenic enzymes (Fig. 6a), in addition to the first steps from rTCA (Fig. 5, pink boxes). PY showed the highest richness of complex carbohydrate breakdown genes and CAZy enzymes, along with ribose and xylose oxidation to G3P by non-oxidative PPP (Fig. 4k). PY presented 22 from 45 CAZy enzymes identified (Table S4).

We found exchange compounds between all MAGs (Table 4), with flux profiles as expected to the syntrophic community from reduced environments, such as mangrove sediment [3]. Besides the exchange compounds, the reactions with higher flux were also in agreement with the functional profile described according to the KEGG database. However, the metabolic models based on the ModelSEED pipeline followed by FBA were important to confirm and expand the KEGG profile, increasing the accuracy of the results and highlighting the importance of using more than one functional database. Moreover, the reconstruction starting from gene content allowed the construction of different media compositions, trying to mimic the environmental fluctuations undergone by mangrove microbial communities. This resulted in a proven effective approach to capture key members and the syntrophic metabolic components according to the environmental context. Recent studies have suggested combining GEMs and FBA to investigate microbial interaction and identify key community members based on different media compositions [159, 160].

### Biotechnological applications

The ETDI’s most representative metabolic pathways, when observed collectively, resemble the metabolic characteristics of syntrophic bacteria communities described for the anaerobic digestion (AD) process occurring in closed anaerobic digesters for wastewater and organic effluents treatment [140, 148, 161], enhanced biological phosphorus removal (EBPR) [86, 162, 163] and nitrogen-rich wastewater treatment systems [98, 101, 164].

### Anaerobic digestion

Anaerobic digestion (AD) is a process occurring in either natural environments or in closed anaerobic digesters for waste(water) treatment, waste disposal and resource recovery [138, 140]. The AD process for wastewater and organic effluent treatment mainly occurs through the sequential processes of hydrolysis, acidogenesis, acetogenesis and methanogenesis, which are performed by highly coordinated syntrophic microbial communities [38, 140, 148, 161]. We identified several metabolic processes described for bioreactors performing AD in our ETDI mangrove MAGs. However, instead of H2/acetate consuming methanogenesis, we observed the gene set required for H2-consuming homoacetogenesis, sulfate respiration by DSR, and acetate/propanoate-consuming populations, which may replace methanogens under high sulfate concentration and highly reduced conditions [131, 143, 146, 147].

As described for most AD systems, the first step is the hydrolysis of complex organic materials to sugars, amino acids and long-chain fatty acids. We observed a broad gene set capable of degrading polysaccharides among the no-Pseudomonadales MAGs (Fig. S3). At the same time, Pseudomonadales MAGs showed most genes involved in alkane degradation and beta-oxidation (Fig. 6d and e). In the AD acidogenesis step, sugars are fermented to VFAs, alcohols and H2 [134, 148]. Most no-Pseudomonadales MAGs presented the genes required for hexose oxidation to acetate through glycolysis or PPP, and VFAs interconversion for thermodynamic equilibrium [119, 148, 149]. Evidence for carbon optimization strategies was observed, as the shortcut conversion of xylose to acetate via XPK/Ack (Fig. 4l and m), the shunt to ethanol production from CBB (Fig. 4v, Fig. S4) and the coupling between TCA, glyoxylate cycle and GCS (Fig. 5), showing a diverse set of TCA intermediaries recycle routes. The ethanol production from acetaldehyde was also broadly distributed. At this point, in a closed AD system, the H2 pressure and acetate concentration progressively increase, which are then consumed by hydrogenotrophic and acetoclastic methanogens, respectively, to achieve favorable thermodynamic conditions [140, 143, 144]. In our case, the ETDI MAGs showed sulfate respiration by DSR, and the gene set of several carbon fixation pathways, which may reduce the H2 concentration, enabling the VFAs oxidation [140, 149, 161]. SAOB may syntrophically cooperate with denitrifiers and SRB that use nitrate or sulfate as the electron acceptor, especially under anaerobic alkaline conditions, outcompeting acetoclastic methanogens [144, 161]. Several factors may cause AD process instability or even inhibition, such as the accumulation of ammonia during the treatment of nitrogen-rich wastes, which may affect the whole microbial community, especially the methanogenic populations [146, 149, 165]. The H2/CO2 concentration combined with other factors such as the type of substrate and pH may also shift the H2-consuming populations from methanogens to homoacetogens and SRB [166].

The co-occurrence of propanoate and butanoate pathways is related to the feedback inhibition effect of acetate on the degradation of other short-chain fatty acids (SCFAs) [119, 148, 149]. During the anaerobic digestion in bioreactors, for example, under high H2 pressure, and high concentrations of SCFAs, acetogenesis and SRB are dominant, producing sulfide, formate and acetate [131, 134, 140]. With the progressive decrease of H2 pressure and increasing acetate concentration, propanoate and butanoate are produced from acetate, to counteract the inhibitory effect of the acetate accumulation [140, 148]. Formate has an inhibitory effect over acetate oxidation, so SAOB start acetate degradation after the depletion of formate, becoming dominant [141]. Formate is an alternative electron acceptor, capable of completely replacing CO2/HCO3-, and the occurrence of several formate dehydrogenases broadly distributed among the ETDI MAGs may suggest a syntrophic H2-formate interconversion to keep related intermediaries and sub-products balanced [141]. Utilizing alternative electron acceptors other than CO2 enhances the metabolic flexibility of acetogens in changing environmental conditions [140, 141, 148]. Identifying genes involved in several possible carbon fixation routes among the MAGs is also a relevant aspect since the biotechnological use of autotrophic organisms with high-CO2-dependent and energetically efficient pathways can be advantageous in bioreactors [124]. The absence of complete methanogenesis pathways in our community metabolic reconstruction also draws attention to the shared features with dark fermentative biohydrogen production (DFBHP), a process performed by facultative and obligate anaerobes growing on simple sugars and starch to produce a mixture of hydrogen gas and VFAs [167]. Many factors constrain the hydrogen production in such systems, especially the presence of H2-consuming bacteria as methanogens, homoacetogens and SRB, which may be balanced by the co-culture with different H2-producing acidogens and SAOB/SPOB [140, 146, 167, 168]. Evidence of the co-occurrence between H2-consuming and H2-producing populations was observed among the ETDI MAGs.

### Wastewater treatment systems

Mixotrophic denitrification (also known as autotrophic-heterotrophic denitrification) has been reported as a highly efficient wastewater treatment strategy to treat sulfate and nitrogen-rich effluents, favored by micro-aerobic conditions, allowing integrated sulfate reduction-sulfide oxidation [98–101, 164], already reported for the Proteobacteria phylum [11, 85, 98, 101, 164].

The heterotrophic denitrification reduces nitrate/nitrite to atmospheric nitrogen in the presence of organic matter. In contrast, sulfur-based autotrophic denitrification uses sulfide, sulfur, or thiosulfate as electron donors, and nitrate/nitrite as an electron acceptor, removing nitrogen without organic carbon requirement [98–101, 164]. TH94 showed the gene set required for mixotrophic denitrification (simultaneous desulfurization and denitrification) (Figs. 2 and 3a, b, a and b, Fig. S1), capable of oxidizing thiosulfate and sulfide using nitrate as its electron acceptor and CO2 as its sole carbon source under anoxic conditions [98, 101], sharing most features presented by the facultative anaerobic moderately halophilic chemolithoautotrophic *Thiohalomonas denitrificans* [85]. The TH94 metabolic reconstruction suggests that it may be coupling microbially driven C, N and S cycling processes, an important microbial mechanism of carbon sequestration already described for a chemoautotrophic S oxidizer Gammaproteobacteria from Burkholderiales order in coastal wetlands ecosystems [85, 169].

Together with mixotrophic denitrifiers, the co-occurrence of community members capable of sulfur oxidation, heterotrophic acetogenesis and prevalence of facultative anaerobic metabolic components is essential to the nitrogen removal efficiency in wastewater treatment systems [98–101, 164]. In our study, we observed a similar microbial community functional distribution for the ETDI MAGs. Interestingly, the mangrove fluctuating aerobic and anaerobic conditions may have a similar effect as the observed improvement in the bioreactor performance under micro-aerobic conditions reported by Zhang et al. 2020 [101]​.

Enhanced biological phosphorus removal (EBPR) is another wastewater treatment process in which inorganic phosphate (Pi) is assimilated and stored intracellularly as polyphosphate (PPPi) by polyphosphate-accumulating organisms (PAOs) [86, 162, 163]. During the aerobic phase, the assimilated Pi is converted into PPPi by polyphosphate kinase 1 (Ppk1). The ATP required for this reaction is supplied mainly by the degradation of polyhydroxyalkanoates, especially PHB. Further, the ATP may be supplied by the degradation of propanoate via propanoyl-CoA to succinyl-CoA [86, 162]. During the anaerobic phase acetate and propanoate are stored as PHAs, the ATP required is supplied by PPPi degradation by exopolyphosphatase (Ppx) or polyphosphate kinase 2 (Ppk2), and the reducing power (NADPH) is provided by glycogen degradation and the TCA cycle [86, 162].

We observed several genes involved in the metabolic routes described for the EBPR process, especially among the Gammaproteobacteria MAGs (Fig. 3c). The phosphate-specific transport system (*pst*ABCS) was identified in all MAGs, except PY. The Pi assimilated is then converted to diphosphate (PPi) by ppa inorganic pyrophosphatase [EC:3.6.1.1], present in most MAGs, and then to PPPi by Ppk1 (*ppk*1), mostly identified among the Gammaproteobacteria MAGs. The genes required for acetyl-CoA conversion to 3-hydroxybutyryl-CoA were identified in PO, TH88, DE and AC and subsequent conversion to PHB in PO (Fig. 6g). The PHB may be depolymerized to acetyl-CoA by phaZ [162], identified in both THs (Fig. 6g). The flux profile showed the propanoyl-CoA production from succinyl-CoA via methylmalonyl-CoA in the anaerobic customized media for AL, as expected in the anaerobic EBPR phase [162]. Similarly, the flux through the propanoyl-CoA degradation to succinate and pyruvate via 2-Methylcitrate was observed only for the aerobic propanoate media in SU and PY (Table 5). Interestingly, in the sulfur-associated EBPR, the polysulfide produced from sulfate reduction during the anaerobic phase (DSR + sqr) may be oxidized to sulfate during the aerobic phase, providing extra energy to phosphate uptake and PPPi accumulation [86]. The co-occurrence of PAO, SRB and SOB may allow the coupling biotransformation of phosphorus and sulfur cycling in mangroves, where oxygen and pH gradients influence the bioavailability of inorganic phosphorus and stability of sulfur compounds [72, 81].

The broad distribution of phosphorus metabolism genes and possible coupling to sulfur, butanoate and propanoate metabolism reinforces the ETDI microbial community’s potential to unveil naturally occurring metabolic components and coupling mechanisms desirable for wastewater treatment systems.

## Conclusion

The mangrove community described in this study suggests a syntrophic scenario showing a cooperative, highly coordinated network of connections, orchestrated by the microhabitat’s biotic and abiotic characteristics and physical chemical requirements/pressure. In fact, the mangrove’s intrinsic characteristics confer several challenges for any living being to succeed, and the syntrophic relationships may improve protection and resilience in the face of harsh mangrove environmental fluctuations. Although ETDI is an effluent treatment station where the current mangrove has been restored and is close to the oil refinery, the microbial community biodiversity and metabolic reconstruction do not indicate a significant disturbance. The broad taxonomic coverage, including six phyla, without a clear dominance pattern, and a highly diverse metabolism suggest a stable microbial community. The metabolic profile observed suggests that trade-offs are probably more advantageous than one population dominating the community. The ETDI MAGs showed metabolic components of facultative anaerobic heterotrophic and autotrophic bacteria, which display a great metabolic diversity, and the ability to grow mixotrophically, enabling situations where autotrophic and heterotrophic growth metabolisms can be simultaneously engaged. The most distinctive feature from ETDI sampling point was the absence of methanogens and methanotrophs. The sulfur/methane metabolism dynamics is governed by the tidal regime and a periodic sampling would be necessary to clarify such metabolic pattern. Interestingly when observed together, the most representative metabolic pathways resemble the metabolic characteristics of syntrophic bacteria communities described for the AD process occurring in closed anaerobic digesters for organic effluent treatment. As described for AD, the entire system is entangled by syntrophic relationships spanning from polysaccharide degraders to biogas producing and carbon sink, a similar picture observed among the eleven ETDI MAGs. Methanogenesis, the last AD step, seems to be replaced by ETDI microbial community strategies that tolerate the high sulfate concentration and reduced conditions characteristic of mangroves. In this study, the thermodynamic balance seems to be fulfilled by the H2-consuming homoacetogens (DE, SU), SRB (THs, DE, SU), and sulfur-based autotrophic denitrifiers (TH94).

The identification of several gene sets and metabolic routes similar to those described for nutrient-rich wastewater treatment systems demonstrate the potentiality of the mangrove microbiome to unveil metabolic capabilities with biotechnological applications. We considered that ETDI mangrove microbial communities represent a resourceful microbial model that occurs naturally in the environment.

### Electronic supplementary material

Below is the link to the electronic supplementary material.

Supplementary Material 1

Supplementary Material 2

Supplementary Material 3

Supplementary Material 4

Supplementary Material 5

Supplementary Material 6

Supplementary Material 7

Supplementary Material 8

Supplementary Material 9

## Data Availability

Metagenome-assembled genomes (MAGs) (SAMN38841318 - SAMN38841328) were deposited in DDBJ/ENA/GenBank under the NCBI BioProject PRJNA954358.

## Abbreviations

eDNA: Environmental DNA
MAGs: Metagenome-assembled genomes
GEM: Genome-scale metabolic model
FBA: Flux balance analysis
ETDI: The name of the mangrove sampled, located in the “Baía de Todos os Santos”, state of Bahia, Brazil
SRB: Sulfate-reducing bacteria
VFA: Volatile fatty acids
SOB: Sulfur-oxidizing bacteria
DSR: Dissimilatory sulfate reduction pathway
SOX: Thiosulfate oxidation by SOX complex
DMSO: Dimethyl sulfoxide
DNR: Dissimilatory nitrate reduction
GH: Glycoside Hydrolase CAZy family
PPP: Pentose phosphate pathway
PEP: Phosphoenolpyruvate
PTS: Phosphoenolpyruvate (PEP)-dependent phosphotransferase system
G3P: Glyceraldehyde-3P
TCA: Tricarboxylic acid cycle
PDH: Pyruvate dehydrogenase complex
PFOR: Pyruvate: ferredoxin oxidoreductase complex
KFOR: 2-Oxoglutarate ferredoxin oxidoreductase complex
WLP: Wood-Ljungdahl pathway
CBB: Calvin-Benson-Bassham
PRK: Phosphoribulokinase
GCS: Glycine cleavage system
THF: Tetrahydrofolate
PTA: Phosphotransacetylase
Ack: Acetate kinase
SAOB: Syntrophic acetate oxidizing bacteria
SPOB: Syntrophic propanoate oxidizing bacteria
CO: complete media
CA/CN: Customized aerobic/anaerobic media
AA/AN: Autotrophic aerobic/anaerobic media
GM: Glucose Minimal media
CA2/CN2: Customized aerobic/anaerobic media without amino acids
refglumin: Reference glucose minimal standard media
LAC: C-D-Lactate standard media
AD: Anaerobic digestion process
EBPR: enhanced biological phosphorus removal
PAOs: Polyphosphate-accumulating organisms
PHB: Polyhydroxybutyrate
