# Supplementary figures and images for: Living in mangroves: a syntrophic scenario unveiling a resourceful microbiome
Source: BMC Microbiol. 2024 Jun 28;24:228. doi: 10.1186/s12866-024-03390-6 (PMC11212195; doi:10.1186/s12866-024-03390-6)

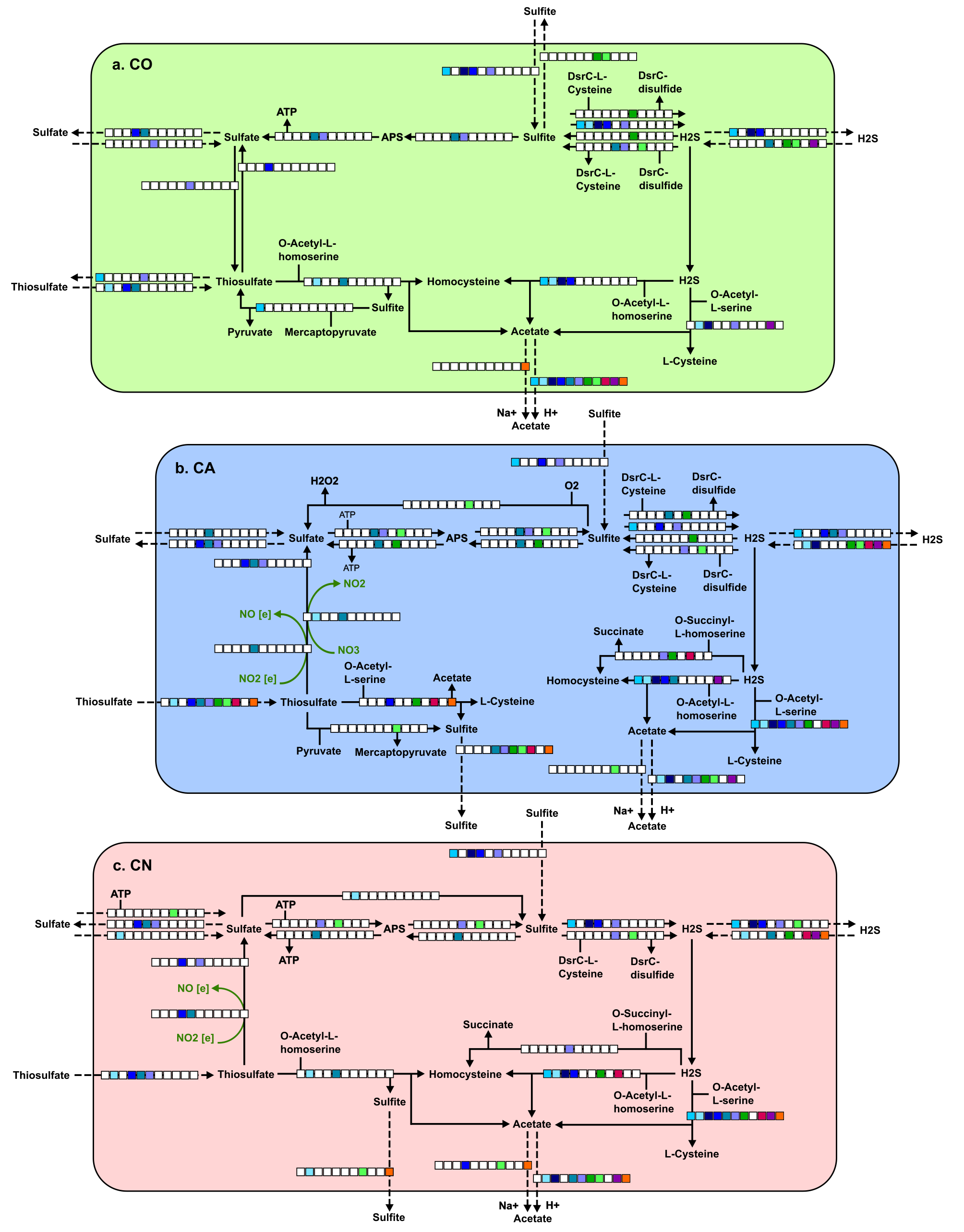

Supplement: Supplementary file 2 — Supplementary Material 2 [file 12866_2024_3390_MOESM2_ESM.png]

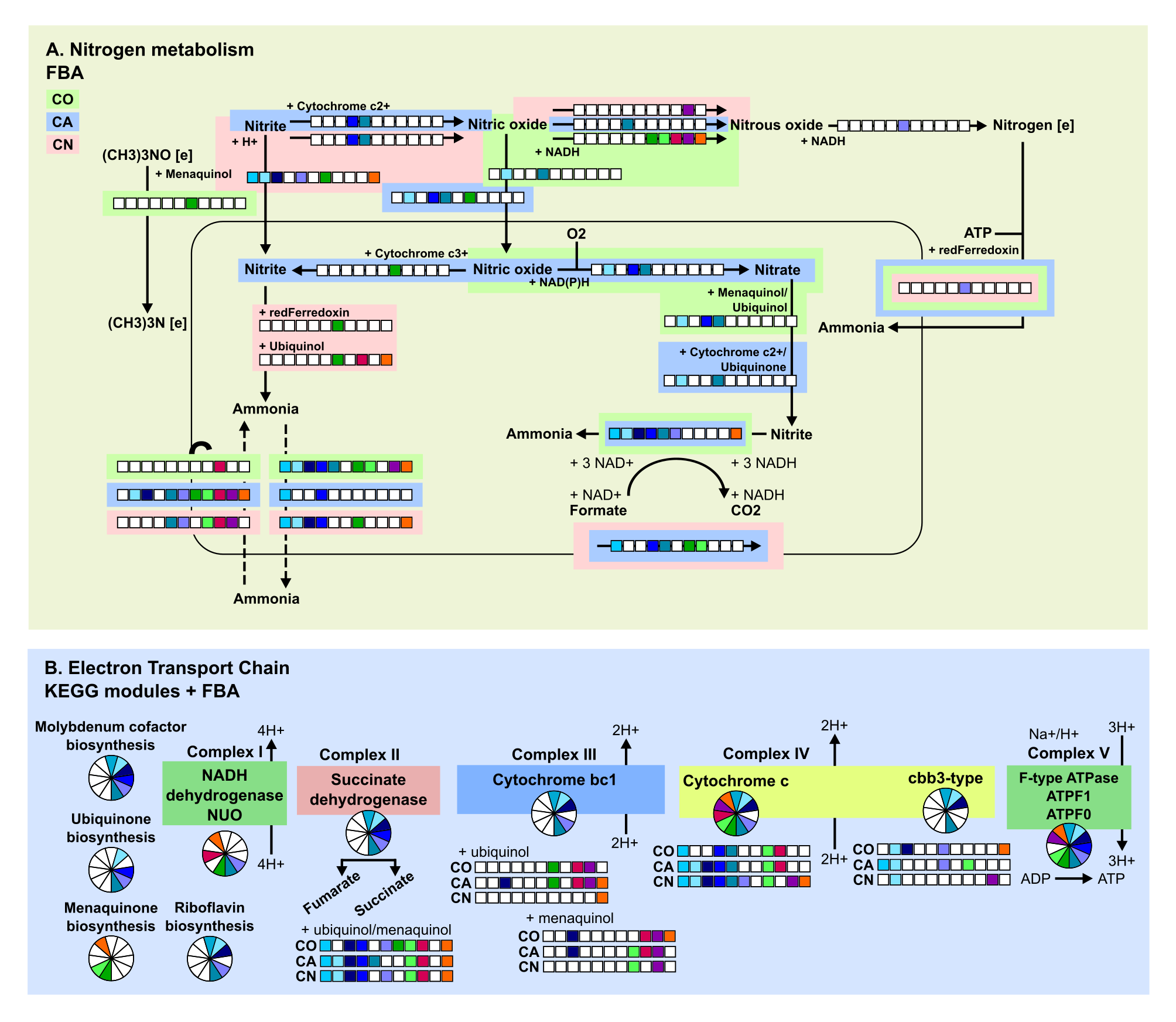

Supplement: Supplementary file 3 — Supplementary Material 3 [file 12866_2024_3390_MOESM3_ESM.png]

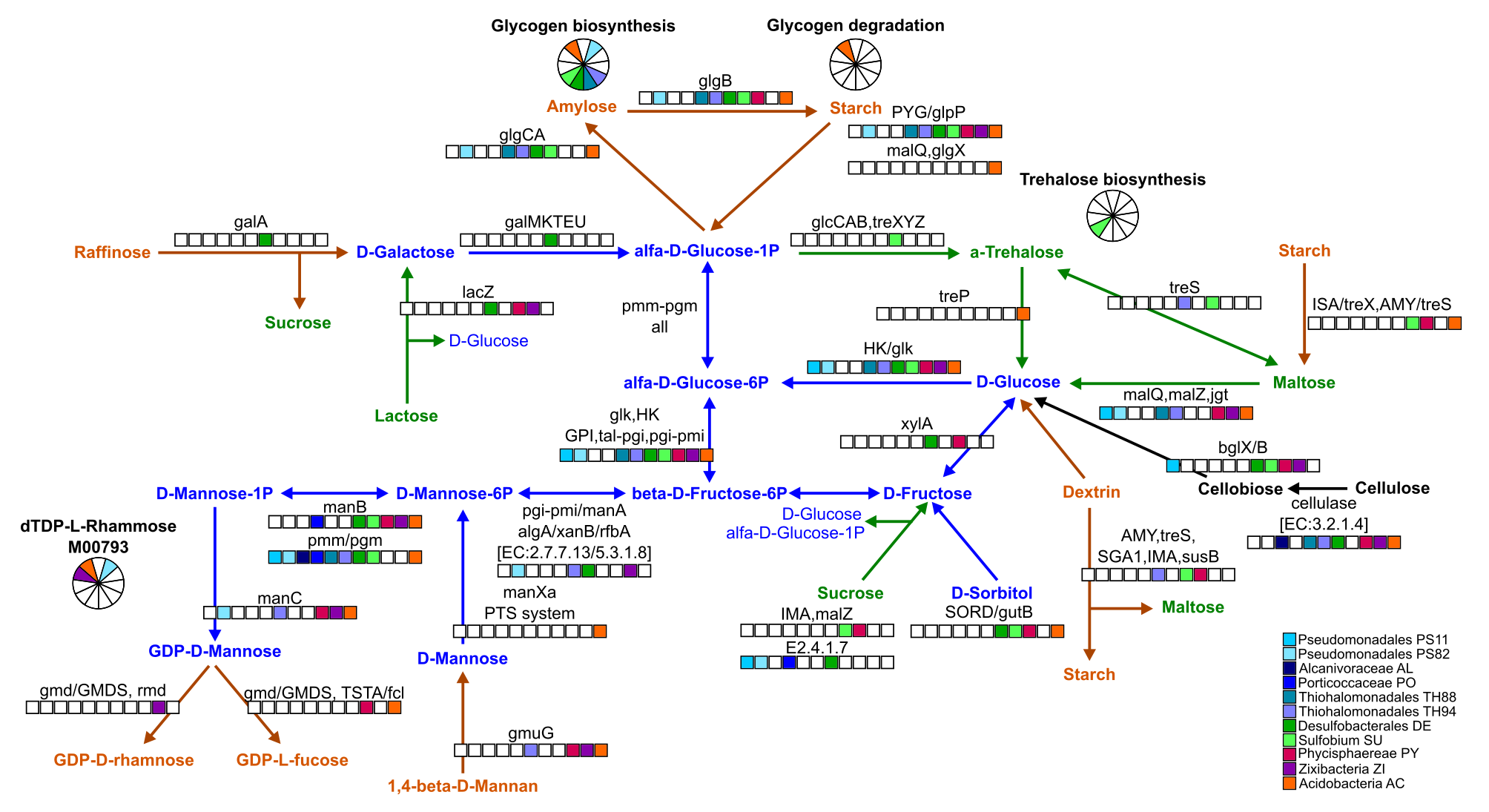

Supplement: Supplementary file 4 — Supplementary Material 4 [file 12866_2024_3390_MOESM4_ESM.png]

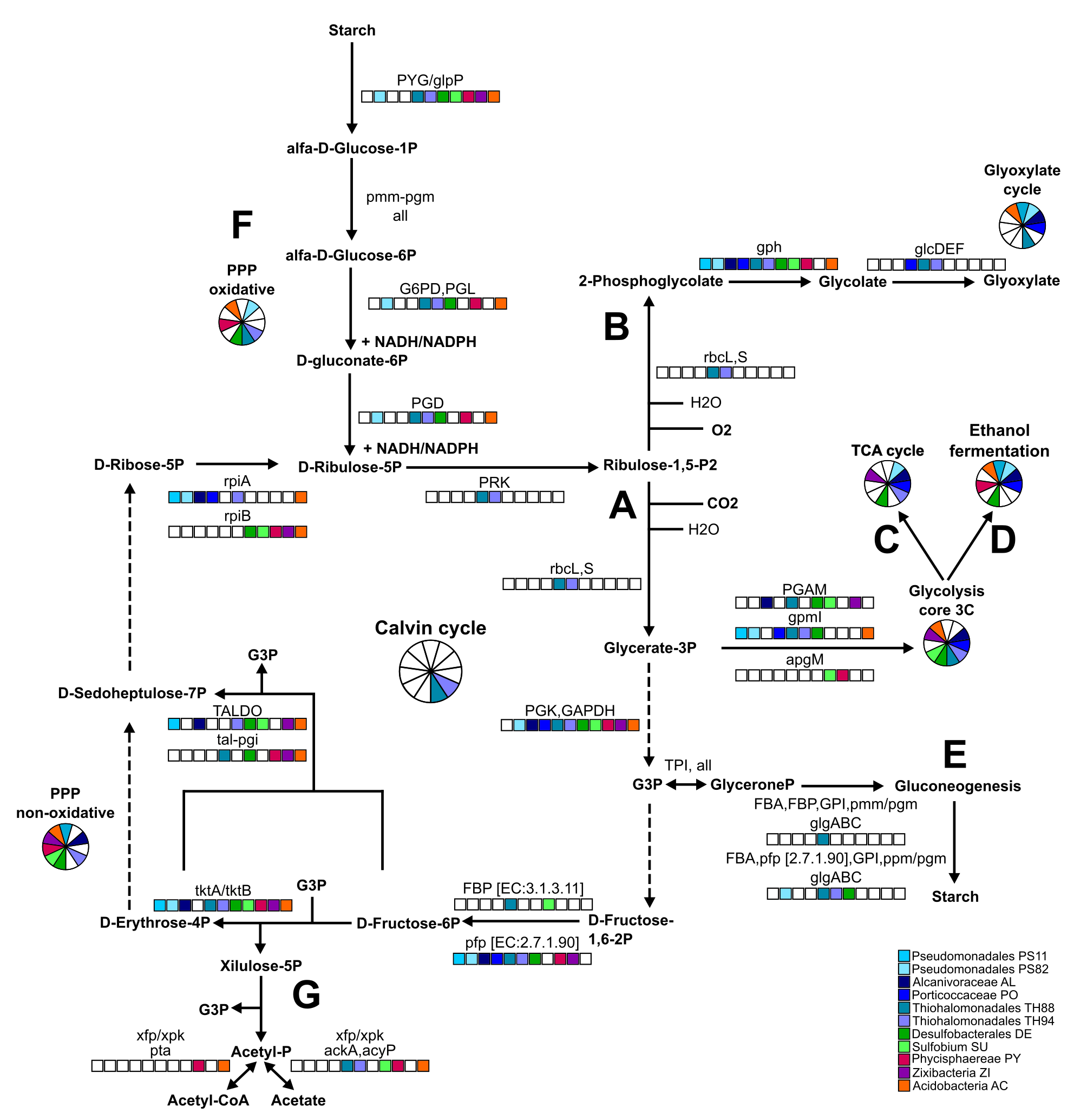

Supplement: Supplementary file 5 — Supplementary Material 5 [file 12866_2024_3390_MOESM5_ESM.png]

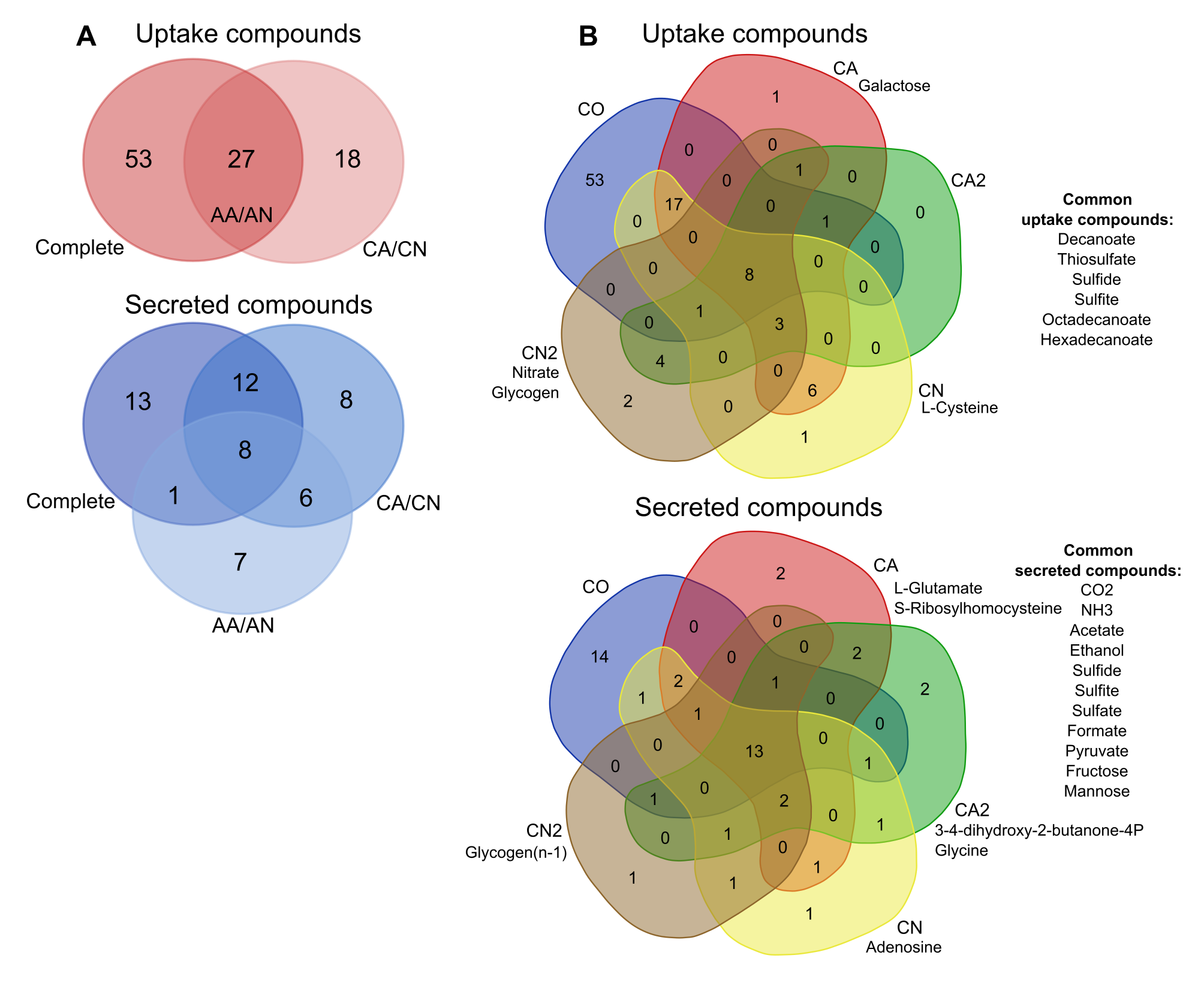

Supplement: Supplementary file 6 — Supplementary Material 6 [file 12866_2024_3390_MOESM6_ESM.png]

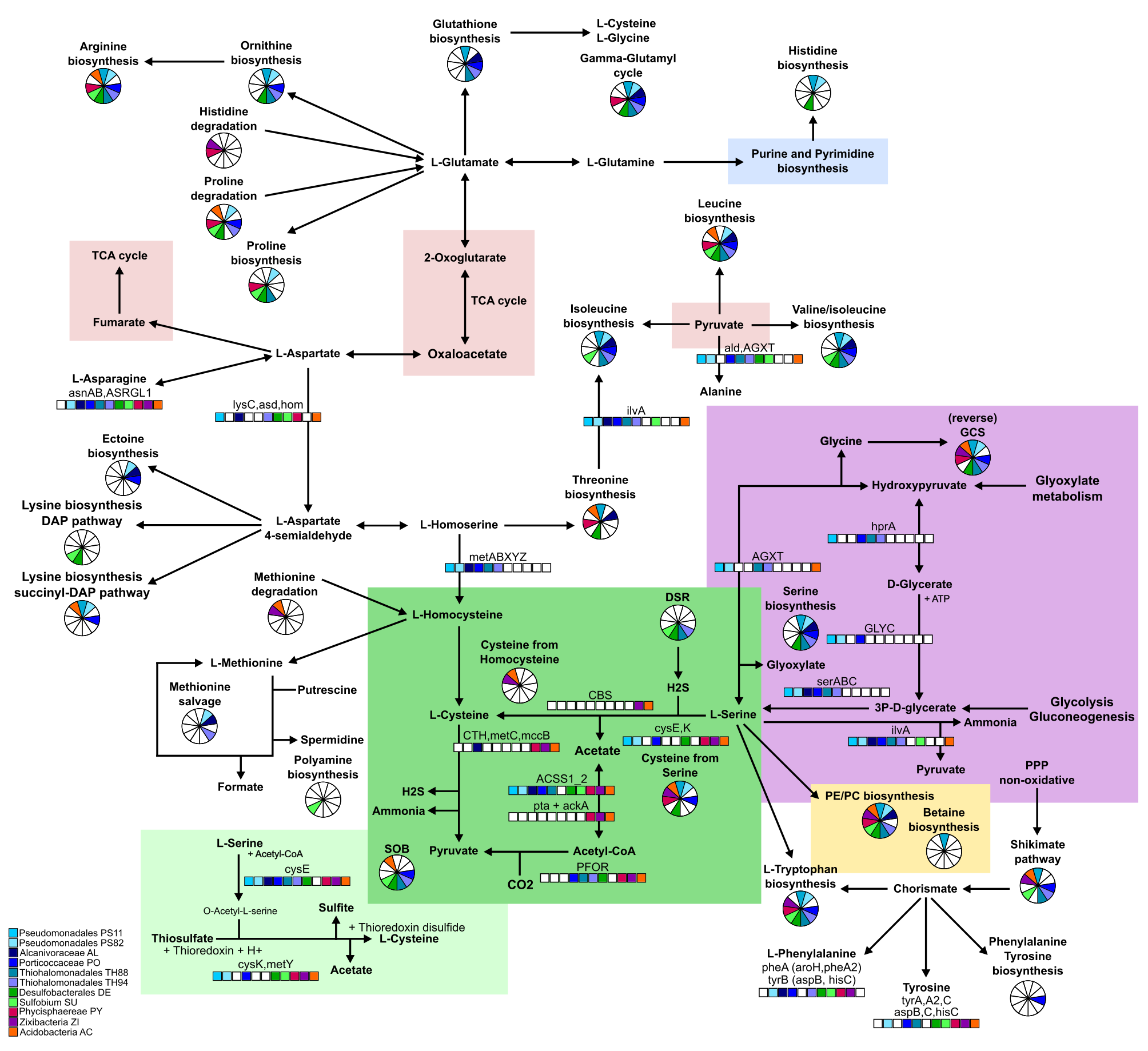

Supplement: Supplementary file 7 — Supplementary Material 7 [file 12866_2024_3390_MOESM7_ESM.png]

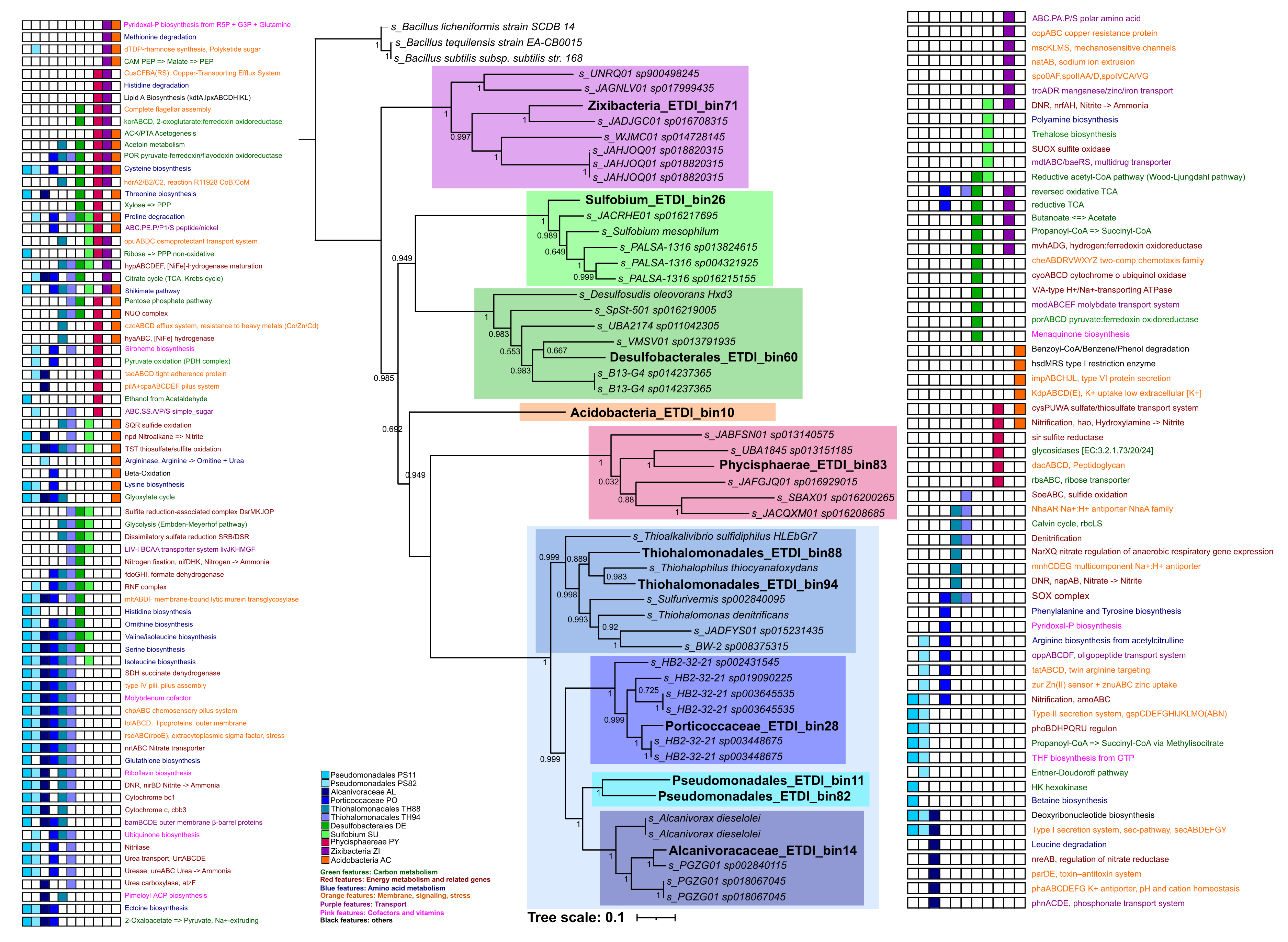

Supplement: Supplementary file 8 — Supplementary Material 8 [file 12866_2024_3390_MOESM8_ESM.png]
