# Supplementary material for: Living in mangroves: a syntrophic scenario unveiling a resourceful microbiome
Source: BMC Microbiol. 2024 Jun 28;24:228. doi: 10.1186/s12866-024-03390-6 (PMC11212195; doi:10.1186/s12866-024-03390-6)
Supplement: Supplementary file 9 — Supplementary Material 9 [file 12866_2024_3390_MOESM9_ESM.docx]

**Living in mangroves: a syntrophic scenario unveiling a resourceful microbiome**

**Supplementary Material**

**Figure legends**

Fig. S1 The sulfur metabolism flux profile comparison between the complete and customized media.

Fig. S2 The nitrogen metabolism flux profile comparison between the complete and customized media.

Fig. S3 Degradation and conversion routes identified for main saccharides. Saccharides and their reactions are indicated by arrows: Polysaccharides (orange), disaccharides (green), and monosaccharides (blue).

Fig. S4 Calvin-Benson-Bassham (CBB) cycle and connections.

Fig. S5. Venn diagrams showing the shared and exclusive compounds between A: complete (CO), customized (CA/CN) and autotrophic media (AA/AN) and B: complete, aerobic and anaerobic customized media with amino acids (CA, CN) and aerobic and anaerobic customized media without amino acids (CA2, CN2). The compounds list for each media is presented in Table S7.

Fig. S6 The amino acid biosynthesis and degradation pathways identified by the KEGG reconstruction.

Fig. S7 Phylogenomic reconstruction of the 11 ETDI MAGs and their evolutionary relationships with public reference genomes. Colored boxes represent the occurrence of that pathway/route/protein in the respective MAG.

**Table legends**

Table S1 Detailed assembly metrics of each MAG.

Table S2 KEGG functional profile: Pathways representativity (number of unique Ko’s) among the ETDI MAGs.

Table S3 KEGG functional profile: Modules occurrence among the ETDI MAGs.

Table S4 List of CAZy enzymes identified according to the KEGG database.

Table S5 Flux Balance Analysis (FBA) profile showing the occurrence of the main reactions from the pathways described. Reaction direction: (-) right to left, (+) left to right.

Table S6 Composition of each media, attempting to simulate the fluctuating ecological conditions characteristic of coastal mangroves.

Table S7 Compounds list for each media.
